# Modern Breast Cancer Surgery 1st Central-Eastern European Professional Consensus Statement on Breast Cancer

**DOI:** 10.3389/pore.2022.1610377

**Published:** 2022-06-15

**Authors:** Zoltán Mátrai, Péter Kelemen, Csaba Kósa, Róbert Maráz, Attila Paszt, Gábor Pavlovics, Ákos Sávolt, Zsolt Simonka, Dezső Tóth, Miklós Kásler, Andrey Kaprin, Petr Krivorotko, Ferenc Vicko, Piotr Pluta, Agnieszka Kolacinska-Wow, Dawid Murawa, Jerzy Jankau, Slawomir Ciesla, Daniel Dyttert, Martin Sabol, Andrii Zhygulin, Artur Avetisyan, Alexander Bessonov, György Lázár

**Affiliations:** ^1^ Department of Breast and Sarcoma Surgery, National Institute of Oncology, Budapest, Hungary; ^2^ Department of Surgery, University of Debrecen, Debrecen, Hungary; ^3^ Bács-Kiskun County Teaching Hospital, Kecskemét, Hungary; ^4^ Department of Surgery, Faculty of Medicine, SZTE ÁOK, University of Szeged, Szeged, Hungary; ^5^ Department of Surgery, University of Pécs, Pécs, Hungary; ^6^ Minister of Human Capacities, Government of Hungary, Budapest, Hungary; ^7^ National Medical Research Radiological Center of the Ministry of Health of the Russian Federation, Russian Academy of Sciences, Moscow, Russia; ^8^ N.N.Petrov National Medical Research Center of Oncology, St. Petersburg, Russia; ^9^ Medical Faculty Novi Sad, Oncology Institute of Vojvodina Sremska Kamenica, University of Novi Sad, Novi Sad, Serbia; ^10^ Department of Surgical Oncology and Breast Diseases, Polish Mother’s Memorial Hospital–Research Institute in Lodz, Lodz, Poland; ^11^ Department of Head and Neck Cancer Surgery, Medical University of Lodz, Lodz, Poland; ^12^ Department of Surgical Oncology, Cancer Center, Medical University of Lodz, Lodz, Poland; ^13^ Clinic of Surgical Oncology, Poznan University of Medical Sciences, Poznan, Poland; ^14^ General and Oncological Surgery Clinic, Karol Marcinkowski University Hospital, Zielona Gora, Poland; ^15^ Plastic Surgery Department, Medical University of Gdańsk/University Hospitals, Gdansk, Poland; ^16^ Department of Surgical Oncology, St. Elisabeth Cancer Institute, Medical Faculty, Comenius University, Bratislava, Slovakia; ^17^ LISOD Hospital of Israeli Oncology, Kyiv, Ukraine; ^18^ National Center of Oncology, Yerevan, Armenia; ^19^ Breast Cancer Department of the LOKOD, N.N.Petrov National Medical Research Center of Oncology, St. Petersburg, Russia

**Keywords:** breast cancer, surgery, consensus statement, oncoplastic surgery, oncology

## Abstract

This text is based on the recommendations accepted by the 4th Hungarian Consensus Conference on Breast Cancer, modified on the basis of the international consultation and conference within the frames of the Central-Eastern European Academy of Oncology. The recommendations cover non-operative, intraoperative and postoperative diagnostics, determination of prognostic and predictive markers and the content of cytology and histology reports. Furthermore, they address some specific issues such as the current status of multigene molecular markers, the role of pathologists in clinical trials and prerequisites for their involvement, and some remarks about the future.

## Introduction

As part of the uptodate multidisciplinary treatment of breast cancer, organ specialized onco-surgery, breast surgery has evolved in many ways over the past decades. The most important causes of this progession are the evidence based clinical science, the biological concept of cancer treatment, the tendency of early diagnosis thanks to populational breast screening programmes and the wide spread of breast cancer awareness, the technological advances in diagnosis, pathology, molecular genetics, pharmacology, radiotherapy and surgery, the quality assured centralization of breast cancer care, and the increased importance of rehabilitation and quality of life. In breast cancer surgery, the principle of minimally effective treatment instead of maximally tolerable treatment has become basic principle and practice.

Up to date surgical therapy for breast cancer will be determined by increasingly precise diagnostic and tumor localizing methods as well as increasingly effective oncology treatment procedures. Organ preserving surgery in combination with primary systemic treatments and the application of oncoplastic principles have become widespread. Sentinel lymph node biopsy is a primary approach in the surgical treatment of the clinically negative axilla, and the indication for axillary lymph node dissection has further decreased by the contribution of regional radiotherapy, medical treatment and targeted axillary surgery. Hereunder we summarise our recommendations on the surgical treatment of breast cancer based on the content of the fourth Hungarian Breast Cancer Consensus Conference as the first Central Eastern European Consesnsus Statement on Breast Cancer Surgery (1) and considering the latest international studies and professional recommendations (2–9).

## Surgical Treatment of Invasive Tumours

The purpose of surgical treatment is to ensure locoregional tumour control, as well as a precise assessment of the locoregional tumour stage. Besides the clinical stage, the biological behaviour of the tumour should also be considered when choosing surgical treatment. When providing surgical treatment for early-stage breast tumours, breast-conserving surgery should be pursued, if there is no objective contraindication. When planning breast-conserving surgery, the cosmetic results of the procedure, patient’s preference and patient’s future quality of life should also be considered. Without good or acceptable cosmetic outcomes, there is no point in breast conservation (10). The informed patient’s opinion is also always taken into account when choosing optimal type of surgery. For unfavourable tumor to breast volume ratio, or locally advanced disease and/or cases with lymph node metastases, the possibility of neoadjuvant oncology treatment should be considered (see primary systemic treatment).

### Criteria for Breast-Conserving Surgery

• Tumour of clinical stage I or II.
• Tumour size: solitary tumour (T1, T2); favourable ratio of healthy breast tissue/tumour volume, tumour location, optimal resecability. If optimal or acceptable cosmetic results cannot be achieved with conventional breast-conserving surgery, oncoplastic surgery should be considered (see oncoplasty), while taking into account the patient’s prefernces (10). Assessment of breast parenchyma and tumour volume using the digital data from the diagnostic contrast enchanced MRI may help in selecting the type of surgical technique.
• Breast-conserving surgery can also be performed after primary systemic treatment. Neoadjuvant treatment can be used to reduce the size of the primary tumour (downsizing) so that the patient may become a candidate for breast-conserving surgery (see primary systemic treatment).
• Lymph node status: N0, N1, no distant metastases: M0 (relative—oligometastases).
• Appropriate adjuvant radiotherapy is provided and accepted by the patient after adequately informed about the adjuvant treatment.
• Appropriate professional, local radiological background is provided for preoperative tumour marking and localisation, intraoperative specimen mammography or ultrasound scanning.

### Contraindication

• Unfavourable ratio of tumour to breast volume (which does not provide adequate oncological/cosmetic results even with oncoplastic techniques).
• Local recurrence or a new primary tumour after previous breast-conserving surgery (if no additional breast irradiation is possible).
• Extensive and/or multicentric ductal carcinoma *in situ* (DCIS) and invasive tumour (see chapter on DCIS, special considerations).
• Inflammatory breast cancer or mastitis carcinomatosa.
• Multiple malignant lesions (>2 lesions, in different breast quadrants, see special considerations).
• Tumour in a previously irradiated area (if no further irradiation is possible).

### Relative Contraindication

Breast-conserving surgery can be performed under certain conditions:

• Multifocal or multicentric lesions (see special considerations).
• Tumour larger than 50 mm (tumour can be reduced with neoadjuvant treatment and/or it can be removed by oncoplasty and a suitable cosmetic/oncological result can also be achieved).
• Tumour located just under the nipple: for breasts of appropriate sizes, a so-called central quadrantectomy or historicaly: cone resection is possible, with sparing of the nipple-areolar complex, see special considerations: skin involvement (nipple-areolar complex) or negative coring specimen taken from the nipple, cannot be confirmed (intraoperative histological examination). However, presence of axillary lymph node metastases, tumour of grade 3, presence of lymphovascular invasion, and triple-negative or HER2-positive tumour may pose a higher risk.
• Mutation of the BRCA genes or other genes with high penetrancy (PALB2, TP53) mutation (see juvenile breast cancer) (2, 4, 5, 11).
• In cases of BRCA 1, 2 positivity, modern mastectomy as well as prophylactic removal of the contralateral breast should also be considered, with immediate or delayed-immediate reconstruction if required (12).

### Special Considerations for Breast-Conserving Surgery

The success of breast-conserving surgery (i.e., how chances of local recurrence can be minimized and cosmetic outcomes improved) is influenced by several factors. The choice of surgical treatment (breast conservation vs. mastectomy) requires careful consideration and planning in cases of multifocal (MF) or multicentric (MC) breast cancers. In both cases, there are multiple cancer focis in the same breast. In MF cases, there are at least two invasive/*in situ* (DCIS) tumours within the same breast quadrant (or breast lobe), separated by non-involved/healthy breast tissue, while in MC cases, malignant foci are located in different breast quadrants (or breast lobes). Classification is important from a surgical point of view, too: multicentric tumours can usually only be removed via two separate incisions during conventional breast-conserving surgery, while multifocal tumours can be removed through one incision. Nowadays, by choosing the right oncoplastic breast conserving technique and with sufficient surgical experience, and also using precise localization techniques, MF tumours and (less frequently) MC tumours can be removed with an intact margin, should the size of the breast allow. An important prerequisite is an accurate preoperative and/or intraoperative diagnosis, of which contrast enchanced MRI scanning (that may detect new foci) and specimen mammogram/ultrasound are mandatory parts. If these criteria are met, a higher local recurrence rate can be reduced to an acceptable level (13, 14). However, for multifocal or multicentric breast cancers, breast-conserving surgeries cannot be considered routine procedures. In each case, malignant foci detected via imaging techniques should be confirmed by targeted sampling, since malignancy is pathologically confirmed in only 96%, even in cases with the highest probability (BI-RADS 5). Foci suspected of malignancy, but which are not available for biopsy (e.g., in the absence of MRI-guided sampling), should be evaluated by onco-team decision.

### Oncoplastic Breast-Conserving Surgery and Modern Mastectomies

Oncoplastic breast surgery is an essential part of the multidisciplinary treatment of breast cancer, combining oncological and reconstructive surgical techniques with the necessary experience and effectiveness. The aim of oncoplastic breast-conserving surgery is to ensure the best possible cosmetic outcome in addition to oncological radicality, by remodelling the remaining breast parenchyma (volume displacement) or replacing missing ones by autologous flaps or implants (volume replacement). In 2009, oncoplastic breast surgical techniques were endorsed by the profession at the St. Gallen Consensus Conference (15).

Oncoplastic breast-conserving surgery involves oncological surgical procedures that require special surgical and plastic surgical (reconstructive plastic surgery) skills and experience (16). Besides outstanding cosmetic results, it allows removal of up to 20–50% of the breast (Level I and II oncoplastic techniques). Some techniques may require immediate or delayed contralateral symmetrisation. These oncoplastic surgical techniques are able to reduce the rate of microscopically involved surgical margins, their rate of morbidity is not higher than those seen with traditional breast-conserving surgeries, and they neither delay adjuvant multidisciplinary treatments, nor complicate oncological follow-up investigations on the long term. However, compared to traditional breast-conserving surgery, such techniques require a longer surgery time (17, 18).

Accurate marking of the tumour bed with clips is essential in oncoplastic surgery, not only for the purpose of radiotherapy planning, but also for the purpose of any local re-excision.

Overall, the oncological outcomes of oncoplastic surgical techniques are comparable to those of traditional breast-conserving surgeries and mastectomies; however, available long-term oncological outcomes are still with limited evidence (1, 5, 17, 19–22).

Skin-sparing mastectomy (SSM) is a type of mastectomy with removal of the nipple-areolar complex (NAC) and limited removal of periareolar skin with immediate/delayed-immediate breast reconstruction. This method can be primarily used for the surgical treatment of extensive ductal carcinomas *in situ* (DCIS), invasive tumours that do not infiltrate the skin, but located close or in the nipple or NAC, especially for centrally located tumours that deform and invert the nipple and areola or M Paget disease. There are no clear international or national recommendations regarding the absolute or relative indications of SSMs. For pathological assessment, examination of the so-called anterior (skin-facing) resection margin is important.

In nipple-sparing mastectomy (NSM), the entire skin of the breast is spared, while in areola-sparing mastectomy (ASM), the nipple is removed along with the parenchyma (23, 24). Surgeries can usually be performed via an incision made in the inframammary fold or in radial direction with or without periareolar extension (e.g., hockey stick incision, batwing etc.), in combination with immediate/delayed-immediate breast reconstruction. Marking of the direct retromammillary gland area for pathological examination, and intraoperative frozen section or postoperative histological examination of the retro-/intramammillary tissue as a separate specimen is an essential part of the method. If tumour is confirmed by the postoperative histology, removal of the nipple with or without the areola is required, which is most often easily carried out even in an outpatient setting. The indication range of NSM has widened, being oncologically equivalent to SSM, but yielding significantly better cosmetic results if there is careful patient selection and immediate/delayed-immediate reconstruction (Evidence II.B) (6, 23). Skin reducing NSMs (SRNSM) are endorsed surgical techniques with adequate radicality and acceptable morbidities, necessitating special surgical experience (25).

SSM/ASM/NSM surgeries are not surgically equivalent to early or classical subcutaneous mastectomy which was routinely performed by leaving a substantial amount of glandular tissue.

### Surgical Resection Margin

Removal of an invasive tumour is oncologically appropriate only if resection margins also prove to be tumour-free on pathological examination (there are no tumour cells within the ink-stained margin). In addition to unifocal tumours, the above recommendation is also considered acceptable for multifocal tumours, following the St. Gallen Consensus Conference of 2019 (7).

Further extension/increase of an intact resection margin is not justified, nor in young patients (<40 years) either in the presence of an extensive intraductal component, in invasive lobular carcinoma or in tumours with unfavourable biological properties. However, in some individual cases with intact margins, re-excision may be justified as defined above (e.g., in multifocal lobular cancers, where the tumour is significantly larger than assessed during preoperative diagnosis and its foci are very close to the stained surgical margin, though there is no ink on them).

For DCIS, both the American NCCN (National Comprehensive Cancer Network; 4) and the European ESMO (European Society of Medical Oncology) recommend achieving an intact resection margin of 2 mm (4, 6).

Intraoperative specimen mammography or ultrasound scanning may also be used to achieve an intact resection margin. In each case, exact orientation (e.g., lateral, medial, superior) of the removed breast specimen is required. Marking the base and walls of the tumour bed with 7marker clips/markers is essential. Three markers are placed to the base of the tumor bed while other 4 one to the parenchyma pillars/walls (posterior, lateral, medial, superior, inferior margins).

Pathological report (macroscopic, microscopic) should include information on the integrity of resection margins. If resection margins are involved, localization and nature of involvement (invasive or *in situ* foci, focal or broad/massive) should be described in millimeters.

It is also important to compare preoperative and intraoperative imaging and pathological investigations.

If the resection margin is positive, re-excision is required (usually once), or if re-excision is not possible and/or in case of or positive margin in re-excision specimen, mastectomy is recommended. Precise orientation and detailed surgical documentation of the tissue removed during re-excision is required. Description of macroscopic and microscopic surgical margins in the pathology report is also justified. If the posterior resection margin is affected and excision has also removed the fascia of the pectoralis major muscle (which was documented in the surgical description), no additional excision is required, only additional boost radiotherapy to the tumour bed. In addition, classical lobular carcinoma *in situ* (LCIS)/lobular neoplasia within the surgical margin is not an indication for re-excision (2–4, 26). However, both pleomorphic and possibly florid variants of LCIS have poorer biological behavior (27, 28); therefore, microscopical complete excision is recommended when the resection margin is involved (see below).

### Non-Palpable Breast Tumours

For non-palpable breast tumours or lesions, preoperative marking is required in all cases. Both classical hook-wire marking and Radioguided Occult Lesion Localization (ROLL), or any other validated methods (Magseed, SaviScout etc.) are suitable for marking and removing non-palpable malignant or suspected malignant lesions. Ultrasound-assisted breast surgery significantly increases the possibility of tumor-free margins and therefore reduces the risk of reoperations (29–31). Several clinical studies have shown that ROLL (localization of non-palpable lesions) technique allows for a more accurate, cosmetically better excision, and that one-session sentinel lymph node biopsy (SNOLL technique) is easier to perform (29–31). Based on the above, hook-wire marking method could be recommended as a first choice for removal of large microcalcifications (DCIS); radial scars and complex sclerosing lesions, where a sentinel lymph node biopsy is not planned.

For invasive tumours, the ROLL technique is primarily used, as it is also suitable for marking sentinel lymph nodes. During surgery, both the tumour and the sentinel lymph node are removed using a hand-held gamma probe. It is mandatory to mark the tumour bed with clips (at least 7 clips) for the accurate adjuvant radiotherapy. Orientation of the removed specimen and specimen mammography/radiography or ultrasound scanning (see surgical resection margin) are also an essential part of the surgery. When choosing the method (ROLL vs. hook-wire marking or other methods like magnetic seeds etc.), the experience of the team (radiologist, surgeon, pathologist) should also be considered (29–31).

### Surgical Treatment of the Axilla

Axillary surgery continues to play an important role in the treatment of invasive breast tumours (1): it provides information on the stage and prognosis of breast cancer and (2) provides regional tumour control. For early breast cancer, axillary surgery is also consistent with trends towards less extensive surgical treatments.

Following clinical axillary ultrasound scanning (AXUS) and ±aspiration cytology (FNAC) or core biopsy, sentinel lymph node biopsy (SLNB) (evidence 2.a) remains the standard axillary staging method for a lymph node-negative (cN0) breast cancer. This method allows reliable and accurate staging in patients with early breast cancer (1–3) and results in lower morbidity than for conventional axillary lymph node dissection (or axillary block dissection) (ALND). Based on the results of several prospective randomized, multicentre studies conducted over recent years (4, 5, 11–14), the indication for ALND has been narrowed down and axillary radiation therapy has become an accepted therapeutic alternative (under certain conditions) (evidence 2.a) (14, 32).

In concordance with the extensive use of primary systemic therapies (PST) in cN positive cases and with the high rate of becoming cN0 after the effective neoadjuvant systemic treatment new methods of targeted axillary surgical care is on the way of being validated and endorsed. New expressions like the targeted lymph node biopsy (TLNB) have been introduced in the literature, which means the selective removal of initialy metastatic lymph node(s) marked with special clips and markers before neoadjuvant therapy or the phrase of targeted axillary dissection (TAD) which is a combination of TLNB and SLNB (33).

SenTa, a prospective multicenter study, showed that TAD minimizes the false negative rate of SLN after neoadjuvant chemotherapy in patients with node positive breast cancer, but detection rate of clipped lymph node was only 86.9% (34).

The multidisciplinary onco-team should decide on the need for and the nature of further treatments, taking into account the final histological results of the SLNs, the type of surgery, biological behaviour or molecular subtype of the tumour, and the patient’s opinion.

### Technical Considerations for Sentinel Lymph Node Biopsy

SLNB is usually performed in conjunction with removal of the primary tumour. If the breast tumour was previously removed and the presence of an invasive/microinvasive tumour has been subsequently confirmed, a sentinel lymph node biopsy has to be performed in a second session.

Currently, two methods are most commonly used to remove sentinel lymph nodes (6): dye labelling (patent blue) and (7) isotopic labelling (colloidal albumin labelled with ^99m^Tc).

Over the past years, several alternative methods have been introduced for sentinel lymph node biopsy, such as fluorescent marking with indocyanine green (ICG) and magnetic marking with nanocolloids containing iron oxide (superparamagnetic iron oxide, SPIO; see the chapter on new methods for sentinel lymph node biopsy).

Identification rate and sensitivity of the isotopic labelling method is significantly higher than for blue dye labelling. The so-called double labelling is the most sensitive method (the identification rate of lymph nodes is 92% on average, while false negative rate of lymph node identification in less than 7% of cases) (35) and it is therefore currently considered an acceptable standard procedure (36, 37). Dye marking can be used as a salvage method, for example following negative lymphoscintigraphy after ROLL labelling. For isotopic labelling, especially in the case of repeated SLNB performed after previous axillary intervention, it is also important to perform a preoperative lymphoscintigraphy to evaluate the projection of sentinel lymph nodes and lymphatic drainage. During an SLNB procedure, in addition to the active lymph node(s) accumulating the isotope, any palpable, non-accumulating lymph nodes that are suspected to be metastatic lesions should also be removed and accurately labelled as non-SLN lymph nodes for the pathologist.

Removal of sentinel lymph nodes adjacent to the internal mammary artery is possible; staging can be refined with this procedure, but the result has little effect on further treatment; its routine use is therefore not justified (32).

#### Indication for Removal of Sentinel Lymph Nodes

• T1-T2 tumours.
• Clinically and radiologically (US) negative axilla, (there are no axillary lymph nodes suspicious of metastasis, or, if present, suspicion is not confirmed by evaluable (non-C1) pathological examination (guided aspiration cytology or core biopsy).
• After neoadjuvant (primary systemic) treatment (PST) if presence of axillary metastases was not confirmed prior to treatment.

#### Sentinel Lymph Node Biopsy in Other Special Cases

• Multicentric and multifocal lesions (20).
• Tumour size T3.
• After previous axillary surgery or breast augmentation.
• Male breast cancer.
• During pregnancy, using a low-dose (≤10 MBq) isotope (dye labelling is contraindicated in pregnancy).
• And after neoadjuvant systemic treatment, if regression, down-staging has occurred as a result of the treatment (cN positivity was turned to ycN0) (see “Neoadjuvant treatment” for details) (20).

#### Contraindication

• Inflammatory breast cancer.
• T4, tumours of stage 4.
• Lymph node metastasis confirmed by other methods [e.g., clinically/radiologically (PET CT) highly suspected axillary lymph node/s; ultrasound-guided FNA/core biopsy].
• Known allergic reaction to markers.

### Axillary Lymph Node Dissection

During ALND, at least ten lymph nodes at axillary levels I and II should be removed, sometimes including also level III (5, 33–38). There are no clear international recommendations for the removal of lymph nodes at axillary level III, performable in cases of resectable Level III metastatic node/s, or in cN2 cathegory. Their removal does not significantly affect either disease-free or overall survival (20, 33).

If technically possible, branches of intercostobrachial nerve should be preserved, which results in reduced rate of postoperative pain and numbness in the upper limb (4).

#### Indication for Axillary Lymph Node Dissection

• concomitantly with surgical treatment of invasive breast cancer if preoperative clinical investigations (ultrasound-guided FNAC/core biopsy) have confirmed the presence of axillary lymph node metastases.
• After SLNB, if there is metastasis in >2 SLNs (macrometastases) and/or the patient does not meet selection criteria for study Z-0011 (38) [clinically negative (physical examination, AXUS, FNAC) axillary lymph nodes, breast-conserving surgery, up to two positive SLNs (micro/macrometastasis, macroscopic extracapsular tumour spread, lymph node conglomerate, neoadjuvant treatment), whole breast irradiation + adjuvant systemic treatment].
• Mastectomy and SLNB, if no postoperative radiotherapy is planned and the SLN (even if only one single lymph node) contains macrometastasis.
• If ultrasound-guided FNAC/core biopsy performed before neoadjuvant (primary systemic) treatment confirms lymph node metastasis and AXUS continues to report suspected lymph nodes after PST; concomitantly with breast surgery.
• Or if SLNB performed after neoadjuvant (primary systemic) treatment confirms axillary lymph node macrometastasis; concomitantly with or after breast surgery. In case of having only isolated tumour cells or micrometastases in the SLN/s after PST, the St Gallen Consensus Panel voted 89% and 60% against completional ALND (5).
• In cases of insufficient or no sentinel lymph node/s presentation (no hot spots), either pre- or intraoperatively; in such cases a so-called axillary lymph node sampling or limited axillary lymph node dissection (axillary sampling plus resection of any suspicios axillary lymph node/s) should carried out by removing at least four lymph nodes (up to 6 nodes) optimaly located at level I of the axilla. Criteria for this intervention are: invasive tumours confirmed by core biopsy; preoperative axillary ultrasound did not confirm suspect lymph nodes; and no nodules suspect of being enlarged metastases are observed during surgery. DCIS (no confirmed invasive/microinvasive parts), neither ALND nor sampling is required (33).

#### ALND Can Be Omitted

If clinically (AXUS negative, in cases of uncertainty AXUS-guided FNAC/core biopsy is negative) the result of disease assessment and SLNB (evidence 2.a) is cN0 (2–4, 20)

• pN0 (sn), i.e., no metastases in the sentinel lymph node(s).
• pN0 (i+) (sn), i.e., SLN involvement of ITC (isolated tumour cell) category can be confirmed.
• pN1mi (sn), i.e., SLN contains at most micrometastases.
• pN1a (sn), if only 1 to 2 SLNs are metastatic (macrometastases), the patient meets the inclusion criteria for study Z-0011 (38). If a clinically positive lymph node is confirmed at the time of diagnosis (US-guided FNAC/core biopsy has confirmed axillary lymph node metastasis) and regression, down-staging occurs as a result of primary systemic treatment, then the result of performed SLNB is ypN0 (sn), i.e., no metastases are present in the sentinel lymph node(s), and ALND may also be omitted. To reduce the rate of false negative results, at least three sentinel lymph nodes must be removed in such cases, and double labelling is mandatory, pretreatment metastaic lymph node marking is highly recommended. If fewer (1, 2) SLNs are removed, ALND can be replaced by axillary radiotherapy (36, 37).
• For mastectomy, if only 1–2 SLNs are metastatic, ALND can be replaced by axillary radiotherapy (7, 37).

### Intraoperative Assessment of Sentinel Lymph Nodes

Indications for intraoperative assessment of SLNs and the resultant burdens for the patient (longer surgery time) and health care system have decreased significantly with the decreasing indications for ALND (36–40). Based on the new guidelines, and with increasing use of alternative axillary radiotherapy, ALND is indicated in an ever-smaller subgroup of patients (<10%).

Based on new indications for ALND, intraoperative SLN assessment is recommended in the following cases:

• When performing mastectomy, if adjuvant radiotherapy is not planned or not accepted by the patient in advance.
• During surgery following neoadjuvant/primary systemic treatment, if SLNB is performed, with a minimum requirement of removing at least two sentinel axillary lymph nodes for cN0 and three lymph nodes for cN1-ycN0.

## Surgical Treatment of Non-Invasive Tumours (Carcinoma *In Situ*)

*In situ* breast carcinomas include the more common and clinically more significant ductal carcinoma *in situ* (DCIS) and Paget’s disease. The ductal form is now considered a precursor of invasive breast carcinoma. According to the new nomenclature, lobular carcinoma *in situ* (LCIS), which was previously classified into this group, is now called lobular neoplasia and, unlike DCIS, it is considered a non-obligatory precursor of invasive breast cancer, and not a malignant disease. It increases the risk of later breast cancer (RR: 5.4–12), but does not require active treatment. The pleomorphic and florid variant of LCIS may behave similarly to DCIS, so its treatment should be the same (41).

With the spread of populational mammography screening, the incidence of DCIS now exceeds 20% in some countries, compared with an earlier incidence of 1%. In untreated cases, the risk for progressing to invasive carcinoma within 10–20 years from the diagnosis is about 30–50%. Clinical observations suggest that the presence of a high-grade comedo-type DCIS and necrosis, as well as age less than 50 years, indicate poorer biological behaviour and also a higher likelihood of local recurrence. In practice, the so-called Van Nuys Prognostic Index and its improved version, the University of Southern California/Van Nuys Prognostic Index are useful tools. The latter also includes the completeness of surgical excision and the patient’s age (the former did not take age into account) in addition to the size and pathological grade of the lesion, when calculating disease prognosis/recurrence. A separate category is the microinvasive (T1mi) form, which in terms of behaviour is closer to DCIS than to invasive cancers (42); the free 2 mm surgical margin that is adequate for a DCIS will therefore also be optimal here. In this case, a chance of metastasis is already present, but with a significantly lower frequency than in larger invasive tumours; however, SLNB is required. The presence of a microinvasive focus is strongly correlated with the extent of DCIS.

### Diagnosis

This disease is primarily detected on mammography screening in asymptomatic women in the form of calcifications of various sizes and appearances (sensitivity 87%–95%) (43). The increasing use of contrast enchanced MRI scanning may help determine the extent of the disease more accurately, especially in high-grade DCIS, where the sensitivity of the procedure is 73%–100% (43, 44), and this may also support the planning of accurate surgical treatment. This disease is associated with clinical symptoms, such as palpable lumps or nipple discharge, in only 5%–10% of the cases. The preoperative diagnosis with core biopsy (or vacuum-assisted core biopsy (VAB)) is essential, since this will clearly confirm the presence of the disease, and it is also suitable for the detection of possible invasive/microinvasive foci (necessitating axillary staging). If the non-malignant biopsy specimen does not contain calcification, sampling is generally not considered to be representative. In such cases, repeated image guided biopsy (optimaly VAB) should be done, if needed by insuffitient result of the repeated biopsy, image-guided (guided by wire, isotope labelling, radioactive or other magnetic labelling seeds) surgical excision for diagnostic purposes is warranted.

### Surgical Treatment

There is no difference in survival between patients undergoing mastectomy and those undergoing breast-conserving surgery plus adjuvant whole breast irradiation.

Since in most cases the disease is not palpable, different kind of tumour labelling technique (wire hook or isotope labelling method, special seed markers) should be used in such cases to achieve successful surgical treatment (see below).

In case of breast conserving surgery, wide excision with a tumour free surgical margin is essential (26). For DCIS, due to a so-called discontinuous growth pattern, a broader intact safety zone is required, compared to invasive tumours. The NCCN (4) and the ESMO (3) consider that an intact margin of at least 2 mm is optimal. As the chance for local recurrence is higher for excisions with close margin/s (<2 mm), consideration of an additional treatment (re-excision, irradiation, tumour bed irradiation with an additional boost dose) is recommended. A close resection margin direct to the skin or to the chest wall continues to be an exception for re-excision, if the resection included the complete parenhcyma and superficial fascia till the subcutaneous fat and the pectoral fascia towards the posterior has also been removed (43). The presence of classical LCIS in the resection margin does not result in an increased local recurrence rate; in such cases, no additional excision or further surgery is required.

Mastectomy is primarily recommended (relative indication) for multicentric/diffuse and/or large (>50 mm) lesions. In cases when the mammary gland to tumour volume ratio (cosmetic result) is suboptimal one should consider surgical options of oncoplastic breast-conserving surgery or modern mastectomies plus immediate breast reconstruction. *In situ* ductal carcinoma can spread to the nipple via the central ductal branch, which is why SSM or ASM with nipple removal is recommended when choosing a type of modern mastectomy procedure and immediate reconstruction. If DCIS cannot be confirmed pathologically in tissue sample behind or direct from the nipple, NSM may also be performed (23). This surgery also provides a good opportunity for immediate breast reconstruction. There are no international first-level evidence recommendations for this indication (23). On pathological investigation, examination of the anterior resection surface is important.

### Surgical Treatment of the Axilla in DCIS

DCIS is defined as non-invasive, which means that it cannot give rise even to lymph node metastases. However, there are reports in the world literature showing that lymph node metastases may occur in the sentinel lymph node in a low percentage of such cases (<10%) (see below). Based on the above, in selected cases, such as extensive tumour size (>50 mm), in the presence of histologically poorly differentiated comedo necrosis, or microinvasive foci, and if a mastectomy or removal of the axillary extension of the breast is planned, sentinel lymph node biopsy is recommended. In the latter cases, removal of the sentinel lymph node is necessary since if the final histological examination confirms invasive and/or microinvasive foci in the breast, SLNB will be significantly more difficult to perform or with less accuracy.

If preoperative investigations suggest pure DCIS less than 50 mm in size (confirmed on core biopsy), no sentinel lymph node biopsy is required in the same session with the excision. If the final histological befund confirms invasive/microinvasive foci in the specimen, SLNB is recommended in a second session.

### Paget’s Disease

Paget’s disease is an *in situ* carcinoma localized within the skin of the nipple-areolar complex (NAC), with a possibility of having an invasive tumorfoci in the parenchyma in almost 80% of the cases. Further invasive or *in situ* foci without any clinicalor symptoms may often be detected accidentaly in peripherial areas of the breast pranehcyma by diagnostical imagines. Preoperative histological examination [surgical biopsy/full-thickness skin biopsy (punch biopsy)] is extremely important for an accurate diagnosis. Similarly, a complex breast imaging, including contrast enchanced breast MRI, is essential for the detection of occult ipsilateral or contralateral lesions. For *in situ* lesions only, the surgical treatment will be local excision with an appropriate tumour free margin and with complete removal of the nipple-areolar complex. If the presence of invasive carcinoma is confirmed, treatment is based on the principles applicable to solid tumours: excision of the central quadrant of the breast, inclusive of the NAC, or mastectomy (with SLNB or ALND; see below). If the invasive tumour is located peripherally, in addition to removal of the NAC, the tumour can be removed by oncoplastic techniques or via a separate skin incision with appropriate axillary staging.

If diagnostic core biopsy confirms other B3 lesions—atypical ductal hyperplasia (ADH), classical lobular neoplasia (LN) (45), flat epithelial atypia (FEA), papilloma (especially if larger than 10 mm, atypical, multiple, peripheral), radial scar, complex sclerosing lesion, phyllodes tumour (PT), atypical or rapidly growing fibroadenoma or large or symptomatic pseudoangiomatous stromal hyperplasia—complete surgical removal is recommended. For B3 lesions (with the exception of ADH and PT), vacuum-assisted biopsy removal and close survaillance are also allowed if necessary technical conditions and experience are met (45).

### Phyllodes Tumour and Sarcomas of the Breast

A tumour of fibroepithelial origin with benign, malignant and borderline forms. Core biopsy is essential for a diagnosis, and if this fails, an excisional biopsy is required, due to the heterogeneity of tumours. Core biopsy does not always result in an accurate diagnostic classification, therefore, cell-rich fibroepithelial lesions will represent category B3 and they should be removed *in toto* (see consensus recommendation on pathology).

#### Surgical Treatment

For a small phyllodes tumour (<5 cm), a wide excision in negative margins (1 cm macroscopic resection margin) without axillary staging will suffice, as this type of tumour may give rise to metastases via haematogenous but not lymphatic spread (except when the presence of axillary lymph node metastasis was confirmed preoperatively). Mastectomy is recommended for extensive lesions (>5 cm) and/or if oncological radicality is uncertain. If mastectomy is performed, immediate breast reconstruction can be carried out. For benign phyllodes tumours, a conservative approach is recommended; close surveillance seems to be sufficient for cases with possible microscopically positive margins, and is also allowed for borderline tumours, judged on individual basis, but in such cases adjuvant radiotherapy is required. For malignant phyllodes tumours, excision in negative margins and adjuvant radiotherapy if the breast is preserved are basic requirements.

In the event of local recurrence, further extensive excision or mastectomy is recommended.

Sarcomas of the breast are rare forming a heterogenous group of malignancies arising from mesenchymal tissues. There are approximately 4.6 new cases per million women per year and account for less than 1% of all breast malignancies (46). The primary sarcoma of the breast is associated with genetic conditions such as LiFraumeni syndrome, familial adenomatous polyposis, and neurofibromatosis type 1. Primary breast sarcomas are also associated with environmental risk factors like arsenic compounds, vinyl chloride, and alkylators. Secondary sarcoma of the breast most often occurs after breast irradiation or other former radiotherapy of intrathoracic malignancies such as nonHodgkin lymphoma. The most common sarcoma of the breast is secondary angiosarcoma. Angiosarcoma of the breast is associated with poor prognosis, and mastectomy is the mainstay of the treatment. In many advanced cases angiosarcoma seems to have a multifocal pattern. Therefore, wide peripheral surgical macroscopic margins of at least 3 cm are recommended.

### Inflammatory Breast Cancer

This is a breast cancer with one of the worst biological behaviours. Its clinical appearance is explained by tumour invasion of the lymphatic vessels of the skin (breast swelling, marked oedema, erythema, peau d’orange), which mimics an inflammatory disease (T4d) (21).

Diagnosis is confirmed based on complex breast examination (US, mammography, MRI if necessary) and histological results (core, punch biopsy), but clinical diagnosis (lymphoedema and erythema involving more than 1/3 of the breast) is essential. At the time of diagnosis, lymph nodes are metastatically involved (N1–N3) in a significant proportion (approximately 80%), and distant metastases can also be detected in almost a quarter of cases. A thorough diagnostics for distant metastases is therefore recommended before starting therapy.

Its treatment primarily is not a surgical indication. Following effective neoadjuvant chemotherapy (and/or targeted therapy), modified radical mastectomy with a view to R0 resection is recommended (3, 4). Sentinel lymph node biopsy (SLNB) is contraindicated in inflammatory breast cancer due to a high false negative rate (of approximately 40%) (47); therefore ALND should be performed. Delayed breast reconstruction can be performed after a negative oncological control, and an appropriate tumour-free period (12 months).

### Gestational Breast Cancer

Gestational breast cancer is breast cancer that occurs during pregnancy or afterwards during breastfeeding (within 12 months). Breast tumour is the most common oncological disease in pregnant women, with an incidence of 1:3000 (48). Diagnosis is usually late, so the prognosis is generally poor.

Treatment should be chosen according to the stage of the disease as in any other case. It should be noted, however, that radiation therapy is contraindicated during pregnancy, but chemotherapy can be administered relatively safely during the second and third trimesters (see Consensus on Systemic Treatment). Pregnancy is not a contraindication to surgery. For breast cancer detected in the first trimester, termination of pregnancy is not justified but should be discussed, and efforts should also be made to avoid preterm birth.

It is recommended that pregnant breast cancer patients are treated in specialy skilled care centres. Surgery can be performed in any trimester. The NCCN (4) recommends performing a mastectomy in the first trimester. In this respect, US and European recommendations differ somewhat (2–5). It should be emphasized that radiation therapy during pregnancy is contraindicated, but if radiation therapy can be postponed until after delivery, breast-conserving therapy does not present any disadvantages compared to mastectomy. However, in the first trimester, mastectomy is recommended due to the significant delay to radiation therapy. Proper axillary staging should be always a part of the surgical treatment. For a clinically negative axilla, sentinel lymph node biopsy may be performed. Use of low-dose isotope (≤10 MBq ^99m^Tc), rapidly followed by surgery and excision of the injection site, after tracer administration, will pose a minimal risk to the fetus, so this can be safely performed during pregnancy as well as in early breast cancer (49, 50). Administration of patent blue is contraindicated. Although large randomized trials cannot be expected due to the low number of cases, experience to date has shown that isotope labelling, with a low dose, can be considered a safe method. According to the St. Gallen recommendation, primary reconstruction with tissue expander after a modern mastectomy (SSM, NSM) is supported, though by a narrow majority; however, longer and more extensive surgery may result in more complications (2).

Breast cancer discovered during breastfeeding is treated according to its stage after cessation of breastfeeding.

### Occult Breast Cancer With Axillary Lymph Node Metastasis

No malignancy/suspected malignancy can be confirmed in the breast with imaging studies (ultrasound, mammography, contrast enchanced MRI) and physical examination, but metastatic lymph node(s) is/are diagnosed in the armpit (by axillary ultrasound, lymph node core biopsy; the breast origin of the metastasis should be confirmed). Less than 0.5% of diagnosed cases are occult breast cancers. In each case, PET CT scanning is recommended to exclude other primary tumours.

Mastectomy (with or without reconstruction) with ALND is one of the available therapeutic options; another option is performing simple ALND followed by breast radiation therapy or other adjuvant oncology treatments. If no mastectomy is performed, some (20%–30%) of the tumours may later become radiologically detectable or symptomatic, and thus removable, therefore close surveillance is extremely important.

### Breast Cancer in Young Women

In current literature, juvenile breast cancer is a term used for breast cancer under the age of 40. This age group does not fall into the age group for mammographic screening, therefore, in the majority of cases (90%) patients present with clinical symptoms. Statistics show that tumours with unfavourable clinicopathological characteristics and that are biologically more aggressive (“triple-negative,” i.e., ER/PR and HER2-negative tumours) are more common below the age of 40. This is also supported by the fact that both recurrence-free and overall survival are lower in this age group (51). For juvenile breast cancer, there is always the possibility of familial, hereditary breast carcinoma. Based on the above, genetic consultation and screening of people carrying *BRCA1* and *BRCA2* mutations is recommended, in an accredited laboratory (2). Newly the St Gallen Consesnus Panel in 2021 stated, if a gene panel testing is chosen, the majority (67%) voted that the preferred panel should routinely include: BRCA1, BRCA2, ATM, BARD1, BRIP1, CDH1, CHEK2, NBN, PALB2, PTEN, STK11, RAD51C and RAD51D, and TP53 genes (5).

Locoregional and systemic treatment should always be individualized, and the principles of surgery do not change in juvenile breast cancer. As a treatment, mastectomy has no advantage over breast-conserving surgery plus radiation therapy in terms of either local recurrence or survival (52).

However, it is recommended that people carrying the mutation be informed in detail in a special centre about the advantages and disadvantages of treatment alternatives, while considering the specific psychosocial, sexual and body image aspects of the situation. The possibility and timing of breast reconstruction should also be addressed when informing the patient. There are several options for surgical treatment. For early breast cancer, breast-conserving surgery with complementary radiation therapy may be performed, if requirements are met. Another proposed alternative treatment is unilateral or bilateral mastectomy (even with immediate reconstruction), which reduces the chances of developing a second breast cancer and also increases disease-free and overall survival, in the long term (53, 54).

### Male Breast Cancer

Its incidence is quite low (male/female ratio 1/100−200), accounting for about 0.2% of malignancies in men. This can be an explanation for the fact that these cancers are detected in a localy advanced stage in most of the cases, and therefore their prognosis is less favourable. Tumour size at the time of discovery is similar to that of female breast cancers, but due to the lack of mammary parenchyma, involvement of the skin and nipple-areola is more common. Diagnostic procedures and staging are the same as for female breast cancers. All men diagnosed with BC should be referred for genetic counselling and, if indicated, *BRCA* mutation testing.

Treatment is also the same as for female breast cancers. From a surgical point of view, the typical central location of the tumour and the low breast tissue to tumour ratio should always be considered. In operable patients, mastectomy and SLNB or ALND when lymph nodes are involved should be the procedures of choice (3, 55). Unlike the volume replacement and aesthetic reconstruction of the female breast, in male cases, it is the primary skin replacement that may represent a challenge for reconstructive surgery.

### Risk-Reducing Mastectomy

Prophylactic bilateral breast removal and breast reconstruction are warranted in high-risk women (carrying certain gene mutations, or who had prior breast irradiation due to lymphoma).

According to the St Gallen Consensus Statement in 2021 the Expert Panel favored consideration of risk-reducing mastectomy for women harboring highly penetrant genes (e.g., BRCA1, BRCA2, TP53, and PALB2), and surveillance with mammography and magnetic resonance imaging (MRI), for women with intermediate penetrance genes (e.g., BARD1, CHEK2, CDH1, and STK11). For women with less penetrant gene mutations (such as ATM, BRIP1, NF1, RAD51C, and RAD51D), the Panel strongly favored surveillance without prophylactic mastectomy (5).

Contralateral risk-reducing mastectomy in patients with breast cancer who carry a genetic mutation may be warranted (evidence 3.b). Up to the age of 80 years, the mean cumulative breast cancer risk of patient carrying *BRCA* mutations is 83% (±7%) for *BRCA1* and 76% (±13%) for *BRCA2*; however, its main feature of this form of the disease is onset at a young age (<40 years) (56). By merely performing bilateral prophylactic mastectomy, the incidence and mortality of breast carcinoma can be reduced by 90%–95% (evidence 3.b) (3, 57).

Gene testing can only be performed in accordance with strict professional standards in accredited laboratories. *BRCA1/2* mutation carriers or other mutations holders with high penetrant genes (see above) should also be informed and various therapeutic options (such as close follow-up, oncopsychological guidance, lifestyle counselling, family screening, reproductive counselling, chemoprevention, and prophylactic mastectomy) should be discussed only in specialized centres with adequate knowledge and experience (21). During genetic testing, *BRCA* mutations are most commonly examined; however, if these are not present and if there is significant family history, other less common genetic disorders should also be considered (Li-Fraumeni syndrome: *p53* mutation; Cowden’s syndrome: *PTEN* mutation; *ATM* mutation; Lynch-syndrome: *MLH1*, *MSH2*, *MSH6*, *EPCAM*, *PMS2* mutation, *RAD51* mutation, *BRIP1* mutation, *PALB2* mutation, *CHEK2* mutation, Peutz-Jeghers syndrome: *STK11* mutation, *CDH1* mutation).

During prophylactic mastectomy, simple mastectomies, SSM, ASM, NSM (evidence 3.c) may be performed as necessary, depending on the patient’s parameters, breast size, and other plastic surgical considerations, with immediate or delayed-immediate breast reconstruction, using biological or synthetic meshes, with expander or silicone implant (evidence 5.c). These surgeries require thorough multidisciplinary preparation, in view of the high-risk group of patients.

Routine sentinel lymph node removal during purely prophylactic surgery is not justified; the chance of occult disease is <5%.

In the United States (58) and to a lesser extent in Europe (57), increasing numbers of women with breast cancer prefer mastectomy, and also request contralateral risk-reducing breast removal. Beneficial effects of bilateral mastectomy on survival if the genetic test is negative have not yet been demonstrated (59, 60, 61). In such cases, careful patient information is also required (2, 3).

## Breast Reconstruction

In a significant proportion of breast cancer patients, complete breast removal is still required for proper oncological surgical care (11, 21, 23, 62). Breast reconstruction is also provided for female patients who have undergone mastectomy. In accordance with European recommendations, when performing mastectomy, the patient must be informed in writing and verbally before surgery about the possibility of breast reconstruction. Indications or contraindications for reconstructive surgery are assessed, and the optimal time for surgery is determined at the mandatory preoperative multidisciplinary breast oncology team meeting (with a plastic surgeon as a member) together with the patient. When reconstruction is requested, the complex treatment plan (in the absence of other contraindications) should take into account the reconstructive surgery, requiring cooperation between the surgeon performing the oncological surgery and the plastic surgeon performing the reconstructive surgery, unless it is performed by a single oncoplastic breast surgeon trained in both areas and with appropriate professional experience. Post-mastectomy breast reconstruction surgery using autologeous flaps may be performed by a plastic surgeon, where minimum professional standards for the procedure are met. Post-mastectomy reconstructive surgery can be performed within one session with tumour removal (immediate reconstruction) or in a delayed version. If oncological treatment has been sufficiently radical to allow immediate/delayed-immediate or two-stage breast reconstruction, SSM, ASM, NSM or SRNSM mastectomy using a state-of-the-art surgical technique is recommended. Oncological results of the latter mastectomies (only those performed with a state-of-the-art surgical technique) are comparable to those of traditional mastectomies. These were professionally endorsed by the St. Gallen Consensus Conference in 2013 (11). Such skin-sparing mastectomies require special expertise and professional experience, and incomplete implementation of these methods results in a significant oncological risk and under-treatment. Skin-sparing mastectomies should only be performed if there is an immediate or delayed-immediate breast reconstruction plan.

Breast reconstruction is a relative indication for surgery, but it is an essential component of the oncological management of breast cancer. It aims to improve quality of life, by acting as one of the most important physical and mental rehabilitation interventions. Breast reconstruction does not delay adjuvant treatment nor affects the treatment outcome, including survival or local control and doesn’t hinder follow-ups. The choice of optimal breast reconstruction technique is the responsibility of the plastic surgeon/oncoplastic breast surgeon, and should be made according to circumstances of the case and the patient’s preferences.

The choice of the optimal breast reconstruction method depends on:

• Patient body type (breast size, obesity).
• Comorbidities (e.g., diabetes) and habits (smoking).
• The type of mastectomy and skin incision (skin-sparing, nipple-sparing).
• The quantity and quality of remaining tissue.
• The plan of multimodal treatment (postoperative radiation therapy or chemotherapy).
• The patient’s mental and physical performance status.
• Surgeon’ Experience.

Depending on when it is performed, breast reconstruction may be:

• Immediate, when reconstruction or some reconstructive steps are performed at the same time of the mastectomy.
• Delayed-immediate, when after SSM,ASM, NSMg, a tissue expander is placed sub- or epipectoral, to bypass the period of adjuvant multidisciplinary treatments, after which reconstruction is completed at a delayed time point using silicone breast implants or autologous flaps.
• Delayed, when one- or multiple-step of breast reconstruction is performed (several months/years) after tumour removal and adjuvant treatment, if there is negative staging.

In recent years, with the broader use of skin-sparing mastectomies, immediate and delayed-immediate breast reconstructions have gained priority, as they have significant cosmetic, psychological, and economic benefits compared to delayed reconstructions.

Immediate or delayed breast reconstruction options after mastectomy:

• Breast reconstruction with autologoustissues:
  ○ With (vascular pedicled or free) flaps transplanted from the abdominal wall or back area (e.g. transverse rectus abdominis (TRAM) or deep inferior epigastric perforator (DIEP) flaps) or the dorsum (latissimus dorsi flap (LD) flap etc.).
    ‐ With local flaps.
• Breast reconstruction with implantation of a tissue expander, especially if adjuvant radiotherapy is planed or had been performed (delayed immediate, or two stage reconstructions) followed by the replacement of definitive silicone implant.
• Breast reconstruction with a silicone implant and a special biological or synthetic mesh (direct to implant techniques) that reinforces the lower pole of the breast (e.g., acellular dermal matrix or various synthetic meshes placed partially subpectoral or prepectoral). The meshes or matrices are crucial in prepecotoral implant-based breast reconstructions (63).
• Breast reconstruction with the combination of autologous tissue (flap) and implant or tissue expander (hybrid reconstructions).
• In cases when post-mastectomy radiation therapy (PMRT) has to be given, the rate of complication of immediate breast reconstructions is increased (capsular contracture, fibrotic transformation of the autologous flap, etc.) If PMRT is given, delayed-immediate (using tissue expander) or delayed breast reconstruction is recommended. The implant placement phase of a delayed-immediate reconstruction or a delayed reconstruction is recommended after complete tissue consolidation or at least 6 months after radiation therapy.
• In case of autologous tissue reconstruction and radiation therapy, the aesthetic outcome of breast reconstruction surgery may be worse than expected, but clinical data are conflicting.
• If a tissue expander or an implant is placed followed by radiation therapy, the rate of early and late complications are significantly higher (capsular contracture, seroma, trophic ulcer).

According to the St Gallen Consensus Statement 2021 with respect to the timing and sequence of reconstruction and postmastectomy radiotherapy, the Expert Panel was completely split about the optimal strategy: delayed reconstruction after radiotherapy 20%, immediate implant in 1 or 2-stage 23%, immediate autologous reconstruction 25%, delayed immediate (expander) 32%—with a large number of abstentions, indicating that there is no established standard with respect to this issue (5).

When tissue reaction (redness, epidermolysis, oedema, etc.) ceases following radiation therapy, possible radiodamaged tissues (e.g., capsular contracure) should be resectedcompletely, or the use of autolgous fat transplantation can promote tissue revascularisation and regeneration. The best functional and aesthetic outcome could be achieved by autologous breast reconstruction. Loss of breast skin can be replaced by local and distal flaps, while the parenchymal volume of the breast can be replaced by implants or autologous flaps. Trends of the last decade have been heading towards implant-based immediate/delayed-immediate reconstructions, since these are with less surgical burden on the patient, the morbidity of the flap donor areais prevented and the patient’s own tissues can be retained for any subsequent salvage interventions.

In patients under age 40 with a cancer family history, genetic testing (BRCA1/2) should be considered before surgery.

When planning a delayed reconstruction, the need for genetic testing should always be considered.

## Primary Systemic (Neoadjuvant) Treatment

A known benefit of primary systemic oncology treatment (PST) is that primarily unresectable tumours may become resectable if they respond well to PST, thereby increasing the rate of breast-conserving surgeries (64, 65, 66). Results reported so far suggest that its effect on disease-free (DFS) and overall survival (OS) is equivalent to that of adjuvant systemic treatment, provided that it is followed by curative surgery and oncology treatment (65). There is also evidence that using neoadjuvant treatment in primary operable cases has no survival advantage over adjuvant treatment, but a minimal increase in the number of locoregional recurrences (evidence 2.a) has been demonstrated (67); it is extremely important to bear this in mind when considering neoadjuvant treatment (6).

Neoadjuvant treatment may be required in patients with stage IIA, IIB, T3N1M0 cancers, where breast-conserving surgery cannot be performed due to unfavourable tumour to breast volume ratio and/or when the patient refuses mastectomy. There is a growing evidence to support the fact that among stage II tumours, primary systemic treatment is worthwhile first of all for ER/PR, HER2-negative (triple-negative) and HER2-positive tumours, when tumour size is larger than 2 cm and/or axillary metastases are present, as well as for ER-positive postmenopausal tumours, where the rate of pathological remission (“down-staging/sizing”) is significantly higher (2–4).

Additional criteria for surgical treatment:

• Core biopsy from the primary tumour and tumour centre labelling (with marker clips/markers).
• FNAC/core biopsy is required in all cases in which axillary lymph node metastasis is suspected clinically and/or on ultrasound scanning.
• Clip marking of the metastatic lymph node is recommended for cases with limited axillary metastatic lymph nodes, in cases in which there is a real chance of cN1− ycN0 (see above TAD).
• MRI scanning is required for treatment monitoring and for designing the final surgical plan, to accurately assess the size and location of the residual tumour (the issue of preserving nipple-areolar complex).
• Indication for neoadjuvant treatment, treatment monitoring and recommendation for subsequent surgical/oncological treatment can only be determined on an individual basis, by the multidisciplinary onco- team.

The choice of the final surgical treatment will depend on the effectiveness of PST, which can be evaluated using complex breast assessment (ideally contrast-enhanced breast MRI) performed before and after systemic treatment. If partial or complete tumour regression is achieved, breast-conserving surgery can be performed often with techniques used to remove non-palpable tumours. Further conditions enabling breast-conserving surgery are as follows: the tumour can be removed with microscopical free surgical margins; no extensive microcalcification suspicios for malignancy demonstrated on mammogram; and an adequate cosmetic result can be achieved with the breast conserving surgery. Surgical excision of the tumour is performed based on the tumour size remaining after the PST, using a marker clip/marker inserted before treatment (2, 67).

For tumours with aggressive biological behaviour (e.g., triple negative, HER 2 positive, grade III, high Ki67) the volume of the breast tissue to be removed should be considered carefully on an individual basis, and the specimen should be large enough to allow an accurate pathological analysis, regardless of the degree of regression (67). Intraoperative specimen radiography/mammographic of the oriented specimen is a prerequisite. Tumour bed should be marked with clips. During surgery, effort should be made to completely remove the microcalcification. There are also data showing that in selected cases, breast-conserving surgery can also be carried out for multifocal and multicentric tumours, if surgical excisions can be performed with a microscopical free surgical margins (2, 68).

### Treatment of the Axilla/Sentinel Lymph Node Biopsy

An axillary SLNB may be performed before initiating primary systemic therapy. Advantages of the method: it provides a more accurate stage assessment; ALND does not need to be performed later, in the event of a negative SLN; and irradiation of the lymphatic region is also not needed. The disadvantage is that the patient undergoes additional surgery before treatment (which means an increased burden on the patient, along with non-negligible costs); in the event of a positive SLN, ALND must be performed even after PST, if the treatment leads to ycN0 status. In half of the cases, this means over-treatment, since as a result of PST, the axillary lymph node metastasis may regress completely (down-staging), and often only the SLN is positive, but other axillary lymph nodes are not. Benefits of SLN biopsy after neoadjuvant treatment: the patient undergoes one single surgery and ALND can be avoided in a significant number of cases, and it also provides an opportunity to evaluate the axillary response to oncology treatment. The disadvantages of this method are that identification rate of the biopsy is lower, while the rate of false negative cases as well as of axillary recurrences is higher. However, based on the results of several prospective randomized studies, reliability of SLNB after neoadjuvant treatment may be enhanced if a double labelling method (isotope + dye) is used and if at least 3 SLNs are removed (69–72). Based on the above and in line with international recommendations, SLNB is the preferred method for assessing axillary status after neoadjuvant treatment (2, 4, 73, 74). The treatment of the axilla in connection with neoadjuvant therapy is summarized below (Table 1). (See above TAD and metastatic lymph node marking before PST)

**TABLE 1:** Surgical treatment of the axilla after neoadjuvant therapy (7, 33).

Baseline Lymph node status	Lymph node status after neoadjuvant therapy	Axillary surgery	Results of Lymph node pathology examination	Complementary axillary intervention	Regional Lymph node irradiation
cN0	ycN0	SLNB	ypN0	No	No
			ypN1	ALND	Yes, if adverse factors*
cN1	ycN0	SLNB* or TLNB (TAD)	ypN0	No	Yes, if adverse factors*
			ypN1	ALND	Yes
cN1	ycN1	ALND	ypN0	No	Yes, if adverse factors*
			ypN1	No	Yes

SLNB: sentinel lymph node biopsy, SLNB*: double labelling, removal of at least 3 SLNs, TLNB: targeted lymph node biopsy (Selective removal of metastatic lymph node(s) marked before neoadjuvant therapy), TAD: targeted axillary dissection (combination of TLNB ans SLNB), ALND: axillary lymph node dissection, AxRT: axillary radiation therapy. *Adverse factors: age <40 years, Grade: 3, triple-negative breast cancer, T3 T4, low tumour regression grade (TRG).

For pN2 pN3, ALND and AxRT are recommended

#### Recommended Treatment

For clinically/ultrasound-positive axilla:

• ALND is required, if the core biopsy/aspiration cytology of the suspected lymph node is positive and if, after neoadjuvant treatment, the lymph node is still positive clinically and/or based on core/aspiration test.
• If the core biopsy/aspiration cytology of the suspected lymph node is negative, a SLNB should be considered prior to PST; if the result is positive, ALND should be performed after PST.
• If the core biopsy/aspiration cytology of the suspected lymph node is negative and no SLNB is performed before PST, it can be performed (with double labelling only) after successful PST (axilla is also clinically negative during surgery); in the event of a pathologically positive SLNB, ALND should be performed in one session (see above new St Gallen Statement in cases of isolated tumor cells and micrometastases).
• If the axilla is clinically positive (cN1) (negative core biopsy/cytology of the suspected lymph node) and becomes clinically negative following neoadjuvant systemic treatment, removal of three or more sentinel lymph nodes is allowed instead of immediate ALND. If all sentinel lymph nodes removed are negative, no additional axillary surgery is required. If less than 3 (1, 2) SLNs were removed, and these were found to be pathologically negative, axillary radiotherapy should be considered (69).
• If the core biopsy/aspiration cytology of the suspected lymph node is positive and ultrasound-guided labeling of the lymph node is possible before neoadjuvant treatment, and the labeled lymph node can be removed after treatment by targeted axillary surgery (TAD), and it is histologically negative together with 1 or 2 additional SLNs, complementary ALND may be omitted in certain cases (see above targeted axillary approaches) (37, 73, 74).
• In patients with baseline cN2 axillary positivity, ALND with regional irradiation should be performed after treatment, regardless of the response to neoadjuvant treatment.

For clinically / ultrasound-negative axilla:

SLNB can be performed both before and after neoadjuvant systemic treatment (after neoadjuvant systemic treatment double labeling, removal of at least 3 SLNs). If fewer than 3 SLNs were removed during SLNB after PST and if these are found to be negative on pathology examination, axillary irradiation should be considered, due to a higher false negative rate.

In case of cN0 before PST, if sentinel lymph node (SLN) cannot be identified after PST either by preoperative lymphoscintigraphy or using intraoperative techniques (dye labelling and/or isotope labelling), four node sampling technique or TAD could be done to prevent overtreatment. In case of macrometastatic lymph node ALND is recommended (see as well ST Gallen 2021 by ypN0 (i+) and ypN1 (mi) (72).

In cases that cannot be classified according to the above suggestions, the multidisciplinary onco-team should decide on the adequate treatment on an individual basis.

## Palliative Surgical Treatment of Breast Cancer

The treatment of advanced breast cancers is complex and involves all disciplines of a multidisciplinary expert team (pharmacology, radiotherapy, and surgical oncology, diagnostic imaging, pathology, gynaecology, psycho-oncology, social work and palliative care) (78, 79). From the very first moment of diagnosis, the patient should be provided with appropriate psychosocial support and supportive treatment, and adequate interventions should be performed according to their symptoms. Actual palliative interventions should be decided individually at a multidisciplinary onco-team meeting level.

Currently, palliative surgical removal of the primary tumour in *de novo* stage IV breast cancers cannot prolong survival, with the exception of cases with bone-only metastases (79, 80). E2108, a randomized trial of surgery in women with *de novo* stage IV breast cancer, showed that breast sugery does not improve overall survival, thereby contradicting the results of multiple observational studies, while prior randomized trials have provided conflicting data (81). According to BOMET MF 14-01 study, timing of primary breast surgery either at diagnosis or after systemic therapy provided a survival benefit similar to ST alone in *de novo* stage IV BOM BC patients. This is the followup study to their randomized trial (82).

Surgery may be considered in selected patients to improve quality of life, but the patient’s opinion should always be taken into account. If surgery is performed, it should aim at radical removal of the primary tumour. In selected cases, where oligometastatic disease and/or low-volume distant metastasis is sensitive to systemic treatments and complete regression occurs, making long-term survival a reality, locoregional curative treatment should be considered.

Several earlier studies suggested that mBC patients may benefit from surgical removal of the primary cancer. Three randomized trials, among them Austrian Breast and Colorectal Cancer Study Group trial 28, however, yielded conflicting results with a Turkish study suggesting a potential benefit of surgery (83).

In ECOG-ACRIN 2108 with mBC without disease progression after 4–8 months of systemic therapy were randomized to continued systemic therapy with or without additional early local therapy (81). The majority of patients had luminal/HER2-negative breast cancer, 37.9% presented with bone-only disease and 53.8% had received upfront chemotherapy. In the overall study population, no difference in terms of OS was observed (HR 1.09; 95% CI 0.80–1.49); in the subset of patients with mTNBC, additional ELT seemed to have a detrimental effect (risk for death HR 3.5; 95% CI 1.16–10.57). Therefore, additional locoregional therapy may not be regarded as a standard component of mBC treatment.

Prospective clinical trials are needed to more accurately assess the oncological value of locoregional treatments for stage IV breast cancers.

Surgery is indicated when prevention and treatment of bleeding, ulceration or infection is targeted, or for hygienic reasons. If mastectomy is required to achieve radical locoregional control, plastic surgery reconstruction may be needed.

## Surgical Treatment of Locoregional Recurrences

### Recurrence After Breast-Conserving Surgery

The rate of recurrence after previous breast-conserving surgery and subsequent radiation therapy is less than 5%, due to multimodal treatment (75). In the event of a recurrence in the breast or a new primary tumour, mastectomy (after having former WBRT) is usually recommended. Depending on the viability of the skin and the time elapsed since irradiation, immediate reconstruction is also possible for cases with R0 resection. Furthermore, particularly good (cosmetic and oncological) results have been published recently with modern skin-sparing mastectomies (75). However, it has also been shown that, under special conditions, repeated breast-conserving surgery may also be justified. According to the St Gallen Consensus Statement 2021 a major change occurred for ipsilateral local recurrence, because the majority of the panel endorsed another breast conservation procedure with radiotherapy, if the lead team is more than 5 years (Expert Panel 63%) (5). Factors that would favour a second breast conservation were defined as: low risk (small, luminal A; 81%); intermediate (5-year) interval since first diagnosis (64%); the panel was split 50:50 on how the issue should be handled in patients for whom re-irradiation is not an option (5).

The most important criteria for this choice are:

• Tumour smaller than 2 cm.
• Solitary lesion.
• Radiation therapy can be repeated with acceptable toxicity (this may be brachytherapy or, if primary APERT has been performed, total breast irradiation may be carried out).
• If explicitly requested by the patient, after adequate information (higher recurrence rate can be expected) (75).

In cases of recurrences developing after mastectomy, a wide excision is recommended (complemented by radiation therapy, if this was not performed previously), if the foci are radical resectable (R0 excision). It may often be necessary to involve a plastic surgeon to achieve proper soft tissue coverage (flaps) of the chest wall.

Treatment of the axilla in cases of breast cancer recurrence (76):

• If SLNB or limited axillary dissection (fewer than ten lymph nodes have been removed) was previously performed and the patient is currently cN0 staged, reSLNB (ALND for positive SLN) or ALND is recommended. In case of or cN+ ALND is the treatment of choice.
• If ALND was carried out previously (more than ten lymph nodes removed) and the axilla is currently clinically negative, axillary surgery is not recommended; however, if it is clinically positive, axillary exploration and removal of the remaining lymph nodes is necessary.
• Contralateral SLNB is recommended if lymphoscintigraphy clearly indicates the presence of sentinel lymph nodes or a hot spot.

Treatment of isolated axillary recurrence:

• ALND after SLNB (with surgical exploration of interpectoral area and of level III).
• Axillary exploration after ALND, removal of recurrent tumour (when R0 resection is possible).

In the case of supra- or infraclavicular recurrence, systemic treatment and radiation therapy are preferred (77).

## Surgical Treatment of Distant Breast Cancer Metastases

Breast cancer with distant metastases or stage IV is a treatable disease, but it is currently considered incurable, with a median overall survival of 3 years and a 5-year survival of 25% (74, 78, 79). Significant improvements in metastatic breast cancer survival have been achieved in recent years.

However, since distant metastases are local manifestations of a systemic disease, removal of the metastasis alone is not sufficient if the above results are to be achieved; this must be part of a multimodal treatment. Additionally, local surgical treatment should only be considered in cases of oligometastases, which means the presence of solitary or up to five metastases, not necessarily in the same organ.

Metastasectomy/radiation therapy, should be based on a multidisciplinary onco- team decision, is most likely to be considered in the following cases:

• Young patient in good general health condition.
• Small tumour volume.
• Long disease-free period.
• Free from local tumour recurrence.
• Feasibility of R0 resection (80).
• Tumour molecular subtype.

Even for unresectable metastases, histological sampling from the metastasis (surgical/non-surgical biopsy) should be sought, since changes in the primary tumour and the receptor status of metastases, as well as the exclusion or identification of a second, unknown primary tumour, may be crucial in the treatment of metastases (81).

### Treatment of Metastases by Organs

#### Liver

Liver metastases of breast cancer are associated with a higher risk of mortality than involvement of any other distant organ (lung, bone, brain). 5-year survival is 3.8–12% (median survival: 4–21 months) (83, 84, 85).

Currently, no high-level evidence for the oncological effectiveness of surgical removal of liver metastases is available. Local treatment of isolated liver metastases may improve survival only in well-selected cases. Patient selection should be performed from a biological perspective by a multidisciplinary onco-team, for well-assessed, histologically confirmed metastases, taking into account tumour molecular subtype (best ER-, HER2-positive tumour), biological behaviour (disease-free interval between the onset of the primary tumour and of the metastasis should be as long as possible), good tumour response to systemic treatments; metastasectomy should be R0; good general condition, burden of surgery as low as possible (laparoscopy, tumour ablation) and low complication rate are important, so that any further postoperative systemic treatment (evidence 5.c) is not delayed.

#### Lungs

The general principles also apply to the resection of lung metastases, but DFS and OS increases in only a small proportion of patients. It is recommended that metastasectomy be carried out via a minimally invasive video thoracoscopic procedure (VATS) (evidence 5.c).

#### Malignant Pleural Involvement

Requires systemic treatment; if confirmed involvement would change the oncological treatment plan, thoracocentesis and cytological analysis of the aspiratum should be considered, although the false negative rate is high (evidence 3.b). Drainage is only recommended in symptomatic cases with clinically significant amount of hydrothorax (evidence 3.a). Insertion of an intrapleural drain or administration of talc and drugs (bleomycin, biological response modifiers) may be helpful (evidence 3.b).

#### Bone

The most common sites of bone metastases are the femur, vertebrae, upper arm, collarbone, and jawbone. Surgery should be considered if there are fractures or an extremely high risk of fracture, which is most often followed by radiation therapy. Pathological fractures of the femur are the most common, followed by pathological fractures of vertebrae and spinal stabilization surgeries due to their risk (evidence 1.a). Neurological symptoms indicative of spinal cord compression are an emergency, warranting neurosurgical or orthopaedic decompression surgery following diagnostic imaging (MRI). If this is not possible, emergency radiation therapy is required (82). Surgical interventions are complemented by targeted radiation therapy and systemic treatment. If there is no risk of pathological fracture, radiation therapy is recommended (evidence 1.a).

#### Brain

10%–30% of patients with metastatic breast cancer will have a brain metastasis, and solitary cerebral metastasis will occur in 10%–20% of patients. According to randomized clinical trials, neurosurgery/metastasectomy or stereotactic radiosurgery is recommended for this group (evidence 1.b). With complementary whole -brain radiation therapy, this reduces the risk of local and complete cerebral recurrence and increases overall survival (evidence 1.c). Surgical or radiosurgical treatment of solitary or multiple brain metastases is recommended, while for unresectable metastases, the latter is considered.

## Issues Relating to Cooperation Between Surgeons and Pathologists

### Storage of Surgical Preparations (Before Delivery to the Pathology Department)

It is advisable to make the surgical preparation available to the pathology department/pathologist immediately after removal (within a maximum of 30–60 min), without formalin fixation and any incision, and to store it at 4°C until delivery. This may also enable tissue bank sampling. If this is not possible, to ensure optimal receptor assessment, it is advisable to start fixation of the fresh preparation in 10% formalin a minimum of five times the volume of the tissue, preferably stored at 4°C (in a refrigerator), and to store samples in a refrigerator at 4°C until delivered to the pathology department. A validated alternative is vacuum packaging and storage at 4°C followed by transport. In addition to tissue structure, these methods provide the best preservation of both receptor proteins and nucleic acids for optimal assessment of predictive biological markers.

### Specimen Orientation

The surgical specimen should be labelled in the operating room, clearly specifying at least three poles, e.g., medial, lateral and superior. Separate marking of the specimen located just behind the nipple is also required in cases of a nipple-sparing mastectomy. The details of orientation should also be recorded by the pathologist in the description.

If intraoperative histological examination of the retroareolar surface or retro/intermammillary specimen is required, the clinical question should be discussed in advance with the pathologist.

The pathologist should be notified if a previously marked (sentinel) lymph node is also removed after neoadjuvant treatment; the presence of a clip in the lymph node, confirmed on intraoperative specimen radiography/mammography and pathological examination, should be recorded in the surgical description so that all previously marked (marked) lymph nodes were removed during SLNB (72, 73).

### Radiological Examination of the Specimen

For tumours that are non-palpable or not clearly palpable, specimen mammography or ultrasound is required to facilitate pathological processing, irrespective of whether breast-conserving surgery or mastectomy is performed. In cases of a neoadjuvant treatment a clip should be placed into the tumour bed in foreward if clinical complete regression is a realistic option, except in cases when extensive microcalcification is remaining after treatment. The resected specimen should also be sent for intraoperative specimen radiography/mammography or ultrasound scanning to confirm removal of the tumour, and also in order that the pathologist be able to find the tumour bed and judge the exact tumour size.

## New Sentinel Lymph Node Biopsy Methods

Over the past years, several alternative methods have been introduced for sentinel lymph node biopsy. Of these, ICG (indocyanine green) fluorescent labelling, among many clinical applications, may also be used to identify axillary sentinel lymph nodes and perform biopsy (86). Studies to date have shown that the rate of sentinel lymph node identification and sensitivity of the method do not differ significantly from radiolabelling, and these values are better when these methods are used in combination. However, obesity and older age will reduce the identification rate (87).

Magnetic marking of the sentinel lymph node with nanocolloid containing iron oxide (superparamagnetic iron oxide (SPIO) may also be used (87). The detection rate of SLNs and sensitivity of the method are equivalent to those of the radioisotope method. Combined application of these methods may improve sensitivity. However, the magnetic carrier enters the liver and spleen and is stored there, which may make subsequent MRI scanning difficult. This procedure cannot be used when metal implants are located close to the region of interest.

Based on the most recent meta-analysis, both methods, when used alone, show better results than blue dye labelling alone and are equivalent to the classic dual, isotope, and blue dye combination (88–90). In institutes where isotope labelling is not possible, the alternative methods presented here are indeed applicable, but, naturally, after proper validation.

This is part 2 of a series of 6 publications on the first Central-Eastern European Professional Consensus Statements on Breast Cancer covering imaging diagnosis and screening (91), pathological diagnosis (92), surgical treatment (present paper), systemic treatment (93), radiotherapy (94) of the disease and related follow-up, rehabilitation and psycho-oncological issues (95).

## Author’s Note

The consensus document contains product placement without the intention of advertising. Each complex molecular test is unique, and although these can be described without indicating their name (for example with the number of genes tested), not everyone will necessarily understand what this refers to. For this reason, and adopting the practice used in some of the source works, the tests are listed under their trade name.

## References

[1] Lázár G Kelemen P Kósa C Maráz R Paszt A Pavlovics G Modern Surgical Treatment of Breast Cancer. 4th Breast Cancer Consensus Conference. Magy Onkol (2020) 64(4):329–46. 33313609

[2] Coates AS Winer EP Goldhirsch A Gelber RD Gnant M Piccart-Gebhart M Tailoring Therapies-Iimproving the Management of Early Breast Cancer: St Gallen International Expert Consensus on the Primary Therapy of Early Breast Cancer 2015. Ann Oncol (2015) 26:1533–46. 10.1093/annonc/mdv221 25939896 PMC4511219

[3] Senkus E Kyriakides S Ohno S Penault-Llorca F Poortmans P Rutgers E Primary Breast Cancer: ESMO Clinical Practice Guidelines for Diagnosis, Treatment and Follow-Up. Ann Oncol (2015) 26(Suppl. 5):v8–v30. 10.1093/annonc/mdv298 26314782

[4] National Comprehensive Cancer Network (NCCN). Guidelines. Breast Cancer. Version 2 (2022). Available at: www.nccn.org (Accessed January 12, 2022).

[5] Burstein HJ Curigliano G Thürlimann B Weber WP Poortmans P Regan MM Customizing Local and Systemic Therapies for Women with Early Breast Cancer: The St. Gallen International Consensus Guidelines for Treatment of Early Breast Cancer 2021. Ann Oncol (2021) 32(10):1216–35. 10.1016/j.annonc.2021.06.023 34242744 PMC9906308

[6] Cardoso F Kyriakides S Ohno S Penault-Llorca F Poortmans P Rubio IT Early Breast Cancer: ESMO Clinical Practice Guidelines for Diagnosis, Treatment and Follow-Up. Ann Oncol (2019) 30:1674. 10.1093/annonc/mdz189 31236598

[7] Burstein HJ Curigliano G Loibl S Dubsky P Gnant M Poortmans P Estimating the Benefits of Therapy for Early-Stage Breast Cancer: The St. Gallen International Consensus Guidelines for the Primary Therapy of Early Breast Cancer 2019. Ann Oncol (2019) 30:1541–57. 10.1093/annonc/mdz235 31373601

[8] Cardoso F Kyriakides S Ohno S Penault-Llorca F Poortmans P Rubio IT Early Breast Cancer: ESMO Clinical Practice Guidelines for Diagnosis, Treatment and Follow-Up. Ann Oncol (2019) 30:1194–220. 10.1093/annonc/mdz173 31161190

[9] Balic M Thomssen C Würstlein R Gnant M Harbeck N . St. Gallen/Vienna 2019: A Brief Summary of the Consensus Discussion on the Optimal Primary Breast Cancer Treatment. Breast Care (2019) 14:103–10. 10.1159/000499931 31798382 PMC6886108

[10] Pukancsik D Kelemen P Újhelyi M Kovács E Udvarhelyi N Mészáros N Objective Decision Making between Conventional and Oncoplastic Breast-Conserving Surgery or Mastectomy: An Aesthetic and Functional Prospective Cohort Study. Eur J Surg Oncol (EJSO) (2017) 43:303–10. 10.1016/j.ejso.2016.11.010 28069398

[11] Goldhirsch A Winer EP Coates AS Gelber RD Piccart-Gebhart M Thürlimann B Personalizing the Treatment of Women with Early Breast Cancer: Highlights of the St Gallen International Expert Consensus on the Primary Therapy of Early Breast Cancer 2013. Ann Oncol (2013) 24:2206–23. 10.1093/annonc/mdt303 23917950 PMC3755334

[12] Narod SA . The Impact of Contralateral Mastectomy on Mortality in BRCA1 and BRCA2 Mutation Carriers with Breast Cancer. Breast Cancer Res Treat (2011) 128:581–3. 10.1007/s10549-011-1479-1 21455666

[13] Nijenhuis MV Rutgers EJ . Conservative Surgery for Multifocal/multicentric Breast Cancer. Breast (2015) 24(Suppl. 2):S96–9. 10.1016/j.breast.2015.07.023 26303986

[14] Gentilini O Botteri E Rotmensz N Da Lima L Caliskan M Garcia-Etienne CA Conservative Surgery in Patients with Multifocal/multicentric Breast Cancer. Breast Cancer Res Treat (2009) 113:577–83. 10.1007/s10549-008-9959-7 18330695

[15] Goldhirsch A Ingle JN Gelber RD Coates AS Thürlimann B Senn H-J . Thresholds for Therapies: Highlights of the St Gallen International Expert Consensus on the Primary Therapy of Early Breast Cancer 2009. Ann Oncol (2009) 20:1319–29. 10.1093/annonc/mdp322 19535820 PMC2720818

[16] Rubio IT Wyld L Esgueva A Kovacs T Cardoso MJ Leidenius M Variability in Breast Cancer Surgery Training across Europe: An ESSO-EUSOMA International Survey. Eur J Surg Oncol (2019) 45:567–72. 10.1016/j.ejso.2019.01.003 30638809

[17] Hennigs A Hartmann B Rauch G Golatta M Tabatabai P Domschke C Long-term Objective Esthetic Outcome after Breast-Conserving Therapy. Breast Cancer Res Treat (2015) 153:345–51. 10.1007/s10549-015-3540-y 26267662

[18] Losken A Dugal CS Styblo TM Carlson GW . A Meta-Analysis Comparing Breast Conservation Therapy Alone to the Oncoplastic Technique. Ann Plast Surg (2014) 72:145–9. 10.1097/sap.0b013e3182605598 23503430

[19] Kelemen P Pukancsik D Újhelyi M Sávolt Á Kovács E Ivády G Comparison of Clinicopathologic, Cosmetic and Quality of Life Outcomes in 700 Oncoplastic and Conventional Breast-Conserving Surgery Cases: A Single-centre Retrospective Study. Eur J Surg Oncol (2019) 45:118–24. 10.1016/j.ejso.2018.09.006 30352766

[20] Association of Breast Surgery at BASO Association of Breast Surgery at BAPRAS Training Interface Group in Breast Surgery Baildam A Bishop H Boland G Oncoplastic Breast Surgery-Aa Guide to Good Practice. Eur J Surg Oncol (2007) 33(Suppl. 1):S1–23. 10.1016/j.ejso.2007.04.014 17604938

[21] Mátrai Z Gulyás G Kovács T Kásler M . Principles and Practice of Oncoplastic Breast Surgery. Budapest, Hungary: Medicina (2019).

[22] Rezai M Knispel S Kellersmann S Lax H Kimmig R Kern P . Systematization of Oncoplastic Surgery: Selection of Surgical Techniques and Patient-Reported Outcome in a Cohort of 1,035 Patients. Ann Surg Oncol (2015) 22:3730–7. 10.1245/s10434-015-4396-4 25672561 PMC4565865

[23] Weber WP Haug M Kurzeder C Bjelic-Radisic V Koller R Reitsamer R Oncoplastic Breast Consortium Consensus Conference on Nipple-Sparing Mastectomy. Breast Cancer Res Treat (2018) 172(3):523–37. 10.1007/s10549-018-4937-1 30182349 PMC6245050

[24] Dorogi B Újhelyi M Kenessey I Ivády G Mátrai Z . Clinicopathological Correlations of Areola‐sparing Mastectomies versus Nipple‐sparing Mastectomies: Analysis of the Oncological and Cosmetic Importance of the Components of the Nipple‐areola Complex. Breast J (2020) 26(10):2081–6. 10.1111/tbj.13957 32613690

[25] Újhelyi M Sávolt Á Fülöp R Mészáros N Mátrai Z . Evaluation of the Medially Pedicled Skin-Reducing Nipple-Sparing Mastectomy as a Standard Mastectomy Technique for Large and Ptotic Breasts. Breast J (2020) 26(11):2276–9. 10.1111/tbj.13985 32725708

[26] Moran MS Schnitt SJ Giuliano AE Harris JR Khan SA Horton J Society of Surgical Oncology-American Society for Radiation Oncology Consensus Guideline on Margins for Breast-Conserving Surgery with Whole-Breast Irradiation in Stages I and II Invasive Breast Cancer. J Clin Oncol (2014) 32:1507–15. 10.1200/jco.2013.53.3935 24516019

[27] Masannat YA Bains SK Pinder SE Purushotham AD . Challenges in the Management of Pleomorphic Lobular Carcinoma *In Situ* of the Breast. Breast (2013) 22:194–6. 10.1016/j.breast.2013.01.003 23357705

[28] Flanagan MR Rendi MH Calhoun KE Anderson BO Javid SH . Pleomorphic Lobular Carcinoma *In Situ*: Radiologic-Pathologic Features and Clinical Management. Ann Surg Oncol (2015) 22:4263–9. 10.1245/s10434-015-4552-x 25893410 PMC4609251

[29] Takács T Paszt A Simonka Z Ábrahám S Borda B Ottlakán A Radioguided Occult Lesion Localisation versus Wire-Guided Lumpectomy in the Treatment of Non-palpable Breast Lesions. Pathol Oncol Res (2013) 19:267–73. 10.1007/s12253-012-9578-9 23065470

[30] Chan BK Wiseberg-Firtell JA Jois RH Jensen K Audisio RA . Localization Techniques for Guided Surgical Excision of Non-palpable Breast Lesions. Cochrane Database Syst Rev (2015) 12:CD009206. 10.1002/14651858.CD009206.pub2 PMC8881987 26718728

[31] Hoffmann J Marx M Hengstmann A Seeger H Oberlechner E Helms G Ultrasound-Assisted Tumor Surgery in Breast Cancer - A Prospective, Randomized, Single-Center Study (MAC 001). Ultraschall Med (2019) 40(3):326–32. 10.1055/a-0637-1725 29975969

[32] Maráz R Boross G Pap-Szekeres J Rajtár M Ambrózay E Cserni G . Internal Mammary sentinel Node Biopsy in Breast Cancer. Is it Indicated? Pathol Oncol Res (2014) 20:169–77. 10.1007/s12253-013-9680-7 23934505

[33] Banys-Paluchowski M Gasparri M de Boniface J Gentilini O Stickeler E Hartmann S Surgical Management of the Axilla in Clinically Node-Positive Breast Cancer Patients Converting to Clinical Node Negativity through Neoadjuvant Chemotherapy: Current Status, Knowledge Gaps, and Rationale for the EUBREAST-03 AXSANA Study. Cancers (2021) 13(7):1565. 10.3390/cancers13071565 33805367 PMC8037995

[34] Kuemmel S Heil J Rueland A Seiberling C Harrach H Schindowski D A Prospective, Multicenter Registry Study to Evaluate the Clinical Feasibility of Targeted Axillary Dissection (TAD) in Node-Positive Breast Cancer Patients. Surg (2020). [Epub ahead of print]. 10.1097/SLA.0000000000004572 33156057

[35] Cserni G . Őrszemnyirokcsomó-biopszia És Axillaris Blokkdissectio Korai Emlőrákban – Algoritmus Magyarázatokkal, Nyitott Kérdésekkel (Sentinel Lymph Node Biopsy and Axillary Block Dissection in Early Breast Cancer – Algorithm with Explanations, Open Questions). Magyar Sebészet (Hungarian Surgery) J (2016) 69:3–12. 10.1556/1046.69.2016.1.2 26901689

[36] Sávolt Á Péley G Polgár C Udvarhelyi N Rubovszky G Kovács E Eight-year Follow up Result of the OTOASOR Trial: The Optimal Treatment of the Axilla – Surgery or Radiotherapy after Positive sentinel Lymph Node Biopsy in Early-Stage Breast Cancer: A Randomized, Single centre, Phase III, Non-inferiority Trial. Eur J Surg Oncol (2017) 43:672–9. 10.1016/j.ejso.2016.12.011 28139362

[37] Donker M van Tienhoven G Straver ME Meijnen P van de Velde CJH Mansel RE Radiotherapy or Surgery of the Axilla after a Positive sentinel Node in Breast Cancer (EORTC 10981-22023 AMAROS): A Randomised, Multicentre, Open-Label, Phase 3 Non-inferiority Trial. Lancet Oncol (2014) 15:1303–10. 10.1016/s1470-2045(14)70460-7 25439688 PMC4291166

[38] Giuliano AE Ballman KV McCall L Beitsch PD Brennan MB Kelemen PR Effect of Axillary Dissection vs No Axillary Dissection on 10-Year Overall Survival Among Women with Invasive Breast Cancer and Sentinel Node Metastasis. JAMA (2017) 318(10):918–26. 10.1001/jama.2017.11470 28898379 PMC5672806

[39] Horváth Z Paszt A Simonka Z Látos M Oláh V Nagyszegi D Is Intraoperative Touch Imprint Cytology Indicated in the Surgical Treatment of Early Breast Cancers? Eur J Surg Oncol (2017) 43:1252–7. 10.1016/j.ejso.2017.01.003 28139361

[40] Sabel MS . The Need for Axillary Lymph Node Dissection in T1/T2 Breast Cancer Surgery-Counterpoint. Cancer Res (2013) 73:7156–60. 10.1158/0008-5472.can-13-2094 24347232

[41] Kuba MG Murray MP Coffey K Calle C Morrow M Brogi E . Morphologic Subtypes of Lobular Carcinoma *In Situ* Diagnosed on Core Needle Biopsy: Clinicopathologic Features and Findings at Follow-Up Excision. Mod Pathol (2021) 34:1495–506. 10.1038/s41379-021-00796-9 33824462 PMC9595593

[42] Sopik V Sun P Narod SA . Impact of Microinvasion on Breast Cancer Mortality in Women with Ductal Carcinoma *In Situ* . Breast Cancer Res Treat (2018) 167:787–95. 10.1007/s10549-017-4572-2 29119353

[43] Kuerer HM Smith BD Chavez-MacGregor M Albarracin C Barcenas CH Santiago L DCIS Margins and Breast Conservation: MD Anderson Cancer Center Multidisciplinary Practice Guidelines and Outcomes. J Cancer (2017) 8:2653–62. 10.7150/jca.20871 28928852 PMC5604195

[44] Kuhl CK Schrading S Bieling HB Wardelmann E Leutner CC Koenig R MRI for Diagnosis of Pure Ductal Carcinoma *In Situ*: a Prospective Observational Study. The Lancet (2007) 370:485–92. 10.1016/s0140-6736(07)61232-x 17693177

[45] Rageth CJ O’Flynn EAM Pinker K Kubik-Huch RA Mundinger A Decker T Second International Consensus Conference on Lesions of Uncertain Malignant Potential in the Breast (B3 Lesions). Breast Cancer Res Treat (2019) 174:279–96. 10.1007/s10549-018-05071-1 30506111 PMC6538569

[46] Lee JS Yoon K Onyshchenko M . Sarcoma of the Breast: Clinical Characteristics and Outcomes of 991 Patients from the National Cancer Database. Sarcoma (2021) 2021:8828158. 10.1155/2021/8828158 33542674 PMC7843167

[47] van Uden DJP van Laarhoven HWM Westenberg AH de Wilt JHW Blanken-Peeters CFJM . Inflammatory Breast Cancer: an Overview. Crit Rev Oncol Hematol (2015) 93:116–26. 10.1016/j.critrevonc.2014.09.003 25459672

[48] Guidroz JA Scott-Conner CEH Weigel RJ . Management of Pregnant Women with Breast Cancer. J Surg Oncol (2011) 103:337–40. 10.1002/jso.21673 21337568

[49] Mátrai Z Bánhidy F Téglás M Kovács E Sávolt A Udvarhelyi N Sentinel Lymph Node Biopsy in Gestational Breast Cancer. Orv Hetil (2013) 154:1991–7. 10.1556/OH.2013.29771 24317358

[50] Gropper AB Calvillo KZ Dominici L Troyan S Rhei E Economy KE Sentinel Lymph Node Biopsy in Pregnant Women with Breast Cancer. Ann Surg Oncol (2014) 21:2506–11. 10.1245/s10434-014-3718-2 24756813

[51] Lee H-B Han W . Unique Features of Young Age Breast Cancer and its Management. J Breast Cancer (2014) 17:301–7. 10.4048/jbc.2014.17.4.301 25548576 PMC4278047

[52] Kroman N Holtveg H Wohlfahrt J Jensen M-B Mouridsen HT Blichert-Toft M Effect of Breast-Conserving Therapy versus Radical Mastectomy on Prognosis for Young Women with Breast Carcinoma. Cancer (2004) 100:688–93. 10.1002/cncr.20022 14770422

[53] Szollár A Újhelyi M Polgár C Oláh E Pukancsik D Rubovszky G A Long-Term Retrospective Comparative Study of the Oncological Outcomes of 598 Very Young (≤35 Years) and Young (36-45 Years) Breast Cancer Patients. Eur J Surg Oncol (2019) 45:2009–15. 10.1016/j.ejso.2019.06.007 31189512

[54] Fancellu A Sanna V . High Mastectomy Rates in Young and Very Young Patients with Breast Cancer. Are They Justified? Eur J Surg Oncol (2020) 46:1391–2. 10.1016/j.ejso.2019.12.024 31917089

[55] Patten DK Sharifi LK Fazel M . New Approaches in the Management of Male Breast Cancer. Clin Breast Cancer (2013) 13:309–14. 10.1016/j.clbc.2013.04.003 23845572

[56] Antoniou A Pharoah PDP Narod S Risch HA Eyfjord JE Hopper JL Average Risks of Breast and Ovarian Cancer Associated with BRCA1 or BRCA2 Mutations Detected in Case Series Unselected for Family History: A Combined Analysis of 22 Studies. Am J Hum Genet (2003) 72:1117–30. 10.1086/375033 12677558 PMC1180265

[57] Paluch-Shimon S Cardoso F Sessa C Balmana J Cardoso MJ Gilbert F Prevention and Screening in BRCA Mutation Carriers and Other Breast/ovarian Hereditary Cancer Syndromes: ESMO Clinical Practice Guidelines for Cancer Prevention and Screening. Ann Oncol (2016) 27(Suppl. 5):v103–v110. 10.1093/annonc/mdw327 27664246

[58] Kurian AW Lichtensztajn DY Keegan THM Nelson DO Clarke CA Gomez SL . Use of and Mortality after Bilateral Mastectomy Compared with Other Surgical Treatments for Breast Cancer in California, 1998-2011. JAMA (2014) 312:902–14. 10.1001/jama.2014.10707 25182099 PMC5747359

[59] Güth U Myrick ME Viehl CT Weber WP Lardi AM Schmid SM . Increasing Rates of Contralateral Prophylactic Mastectomy - A Trend Made in USA? Eur J Surg Oncol (2012) 38:296–301. 10.1016/j.ejso.2011.12.014 22305274

[60] Yao K Winchester DJ Czechura T Huo D . Contralateral Prophylactic Mastectomy and Survival: Report from the National Cancer Data Base, 1998-2002. Breast Cancer Res Treat (2013) 142:465–76. 10.1007/s10549-013-2745-1 24218052

[61] Hwang ES Lichtensztajn DY Gomez SL Fowble B Clarke CA . Survival after Lumpectomy and Mastectomy for Early Stage Invasive Breast Cancer. Cancer (2013) 119:1402–11. 10.1002/cncr.27795 23359049 PMC3604076

[62] Kronowitz SJ . State of the Art and Science in Postmastectomy Breast Reconstruction. Plast Reconstr Surg (2015) 135:755e–71e. 10.1097/PRS.0000000000001118 25811587

[63] Potter S Conroy EJ Cutress RI Williamson PR Whisker L Thrush S Short-term Safety Outcomes of Mastectomy and Immediate Implant-Based Breast Reconstruction with and without Mesh (iBRA): A Multicentre, Prospective Cohort Study. Lancet Oncol (2019) 20:254–66. 10.1016/S1470-2045(18)30781-2 30639093 PMC6358590

[64] Fisher B Brown A Mamounas E Wieand S Robidoux A Margolese RG Effect of Preoperative Chemotherapy on Local-Regional Disease in Women with Operable Breast Cancer: Findings from National Surgical Adjuvant Breast and Bowel Project B-18. J Clin Oncol (1997) 15:2483–93. 10.1200/jco.1997.15.7.2483 9215816

[65] van Nes JGH Putter H Putter H Julien J-P Tubiana-Hulin M van de Vijver M Preoperative Chemotherapy Is Safe in Early Breast Cancer, Even after 10 Years of Follow-Up; Clinical and Translational Results from the EORTC Trial 10902. Breast Cancer Res Treat (2009) 115:101–13. 10.1007/s10549-008-0050-1 18484198

[66] Kaufmann M von Minckwitz G Mamounas EP Cameron D Carey LA Cristofanilli M Recommendations from an International Consensus Conference on the Current Status and Future of Neoadjuvant Systemic Therapy in Primary Breast Cancer. Ann Surg Oncol (2012) 19:1508–16. 10.1245/s10434-011-2108-2 22193884

[67] Early Breast Cancer Trialists' Collaborative Group (EBCTCG). Long-term Outcomes for Neoadjuvant versus Adjuvant Chemotherapy in Early Breast Cancer: Meta-Analysis of Individual Patient Data from Ten Randomised Trials. Lancet Oncol (2018) 19(27–39):27–39. 10.1016/S1470-2045(17)30777-5 29242041 PMC5757427

[68] Ataseven B Lederer B Blohmer JU Denkert C Gerber B Heil J Impact of Multifocal or Multicentric Disease on Surgery and Locoregional, Distant and Overall Survival of 6,134 Breast Cancer Patients Treated with Neoadjuvant Chemotherapy. Ann Surg Oncol (2015) 22:1118–27. 10.1245/s10434-014-4122-7 25297900

[69] Kuehn T Bauerfeind I Fehm T Fleige B Hausschild M Helms G Sentinel-lymph-node Biopsy in Patients with Breast Cancer before and after Neoadjuvant Chemotherapy (SENTINA): a Prospective, Multicentre Cohort Study. Lancet Oncol (2013) 14:609–18. 10.1016/s1470-2045(13)70166-9 23683750

[70] Boileau J-F Poirier B Basik M Holloway CMB Gaboury L Sideris L Sentinel Node Biopsy after Neoadjuvant Chemotherapy in Biopsy-Proven Node-Positive Breast Cancer: the SN FNAC Study. J Clin Oncol (2015) 33:258–64. 10.1200/jco.2014.55.7827 25452445

[71] Boughey JC Suman VJ Mittendorf EA Ahrendt GM Wilke LG Taback B Factors Affecting sentinel Lymph Node Identification Rate after Neoadjuvant Chemotherapy for Breast Cancer Patients Enrolled in ACOSOG Z1071 (Alliance). Ann Surg (2015) 261:547–52. 10.1097/sla.0000000000000551 25664534 PMC4324533

[72] Galimberti V . Feasibility of sentinel Node Biopsy in Breast Cancer after Neoadjuvant Treatment. Breast (2015) 24(Suppl. 1):PG 9.02. 10.1016/s0960-9776(15)70035-4

[73] Simons JM van Nijnatten TJA van der Pol CC Luiten EJT Koppert LB Smidt ML . Diagnostic Accuracy of Different Surgical Procedures for Axillary Staging after Neoadjuvant Systemic Therapy in Node-Positive Breast Cancer. Ann Surg (2019) 269:432–42. 10.1097/sla.0000000000003075 30312200 PMC6369968

[74] Cardoso F Senkus E Costa A Papadopoulos E Aapro M André F 4th ESO-ESMO International Consensus Guidelines for Advanced Breast Cancer (ABC 4). Ann Oncol (2018) 29:1634–57. 10.1093/annonc/mdy192 30032243 PMC7360146

[75] Walstra CJEF Schipper R-J Poodt IGM van Riet YE Voogd AC van der Sangen MJC Repeat Breast-Conserving Therapy for Ipsilateral Breast Cancer Recurrence: A Systematic Review. Eur J Surg Oncol (2019) 45:1317–27. 10.1016/j.ejso.2019.02.008 30795956

[76] Ishitobi M Fukui R Hashimoto Y Kittaka N Nakayama T Tamaki Y . pTis and pT1a Ipsilateral Breast Tumor Recurrence Is Associated with Good Prognosis after Salvage Surgery. Oncology (2018) 94:12–8. 10.1159/000480516 29017163

[77] Wapnir IL Price KN Anderson SJ Robidoux A Martín M Nortier JWR Efficacy of Chemotherapy for ER-Negative and ER-Positive Isolated Locoregional Recurrence of Breast Cancer: Final Analysis of the CALOR Trial. J Clin Oncol (2018) 36:1073–9. 10.1200/jco.2017.76.5719 29443653 PMC5891132

[78] Cardoso F Spence D Mertz S Corneliussen-James D Sabelko K Gralow J Global Analysis of Advanced/metastatic Breast Cancer: Decade Report (2005-2015). Breast (2018) 39:131–8. 10.1016/j.breast.2018.03.002 29679849

[79] Gennari A André F Barrios CH Cortés J de Azambuja E DeMichele A ESMO Clinical Practice Guideline for the Diagnosis, Staging and Treatment of Patients with Metastatic Breast Cancer. Ann Oncol (2021) 32:1475–95. 10.1016/j.annonc.2021.09.019 34678411

[80] Kwapisz D . Oligometastatic Breast Cancer. Breast Cancer (2019) 26:138–46. 10.1007/s12282-018-0921-1 30324552

[81] Khan SA Zhao F Solin LJ Goldstein LJ Cella D Basik M A Randomized Phase III Trial of Systemic Therapy Plus Early Local Therapy versus Systemic Therapy Alone in Women with De Novo Stage IV Breast Cancer: A Trial of the ECOG-ACRIN Research Group (E2108). J Clin Oncol (2020) 38:LBA2. 10.1200/jco.2020.38.18_suppl.lba2

[82] Soran A Dogan L Isik A Ozbas S Trabulus DC Demirci U The Effect of Primary Surgery in Patients with De Novo Stage IV Breast Cancer with Bone Metastasis Only (Protocol BOMET MF 14-01): a multi-center, Prospective Registry Study. Ann Surg Oncol (2020). Available at: http://link.springer.com/10.1245/s10434-021-09621-8 (In press) 38. 10.1200/JCO.2020.38.18_suppl.LBA2 33532878

[83] Bjelic-Radisic V Fitzal F Knauer FM Steger G Egle D Greil R Primary Surgery versus No Surgery in Synchronous Metastatic Breast Cancer: Patient-Reported Quality-Of-Life Outcomes of the Prospective Randomized Multicenter ABCSG-28 Posytive Trial. BMC Cancer (2020) 20:392. 10.1186/s12885-020-06894-2 32375735 PMC7204290

[84] Khan SA Zhao F Solin LJ Goldstein LJ Cella D Basik M A Randomized Phase III Trial of Systemic Therapy Plus Early Local Therapy versus Systemic Therapy Alone in Women with De Novo Stage IV Breast Cancer: A Trial of the ECOG-ACRIN Research Group (E2108). J Clin Oncol (2020) 38(Suppl. l):LBA2. 10.1200/jco.2020.38.18_suppl.lba2

[85] Ruiz A Wicherts DA Sebagh M Giacchetti S Castro-Benitez C van Hillegersberg R Predictive Profile-Nomogram for Liver Resection for Breast Cancer Metastases: An Aggressive Approach with Promising Results. Ann Surg Oncol (2017) 24:535–45. 10.1245/s10434-016-5522-7 27573523

[86] Motomura K Inaji H Komoike Y Kasugai T Noguchi S Koyama H . Sentinel Node Biopsy Guided by Indocyanin Green Dye in Breast Cancer Patients. Jpn J Clin Oncol (1999) 29:604–7. 10.1093/jjco/29.12.604 10721942

[87] Lin J Lin L-S Chen D-R Lin K-J Wang Y-F Chang Y-J . Indocyanine green Fluorescence Method for sentinel Lymph Node Biopsy in Breast Cancer. Asian J Surg (2020) 43:1149–53. 10.1016/j.asjsur.2020.02.003 32143963

[88] Thill M Kurylcio A Blechmann R van Haasteren V Grosse B Berclaz G The SentiMag Study: sentinel Node Biopsy with Superparamagnetic Iron Oxide (SPIO) *vs*. Radioisotope. Eur J Cancer (2013) 49:S260–S261. 10.1016/j.breast.2014.01.004

[89] Mok CW Tan SM Zheng Q Shi L . Network Meta‐analysis of Novel and Conventional sentinel Lymph Node Biopsy Techniques in Breast Cancer. BJS Open (2019) 3:445–52. 10.1002/bjs5.50157 31388636 PMC6677105

[90] Goonawardena J Yong C Law M . Use of Indocyanine green Fluorescence Compared to Radioisotope for sentinel Lymph Node Biopsy in Early-Stage Breast Cancer: Systematic Review and Meta-Analysis. Am J Surg (2020) 220:665–76. 10.1016/j.amjsurg.2020.02.001 32115177

[91] Forrai G Kovács E Ambrózay É Barta M Bobély K Lengyel Z Use of Diagnostic Imaging Modalities in Modern Screening, Diagnostics and Management of Breast Tumours. 1st Central-Eastern European Professional Consensus Statement on Breast Cancer. Pathol Oncol Res (2022) 220 (3). in press. 10.1016/j.amjsurg.2020.02.001 PMC9214693 35755417

[92] Cserni G Francz M Járay B Kálmán E Kovács I Krenács T Pathological Diagnosis, Work-Up and Reporting of Breast Cancer 1st Central-Eastern European Professional Consensus Statement on Breast Cancer. Pathol Oncol Res (Forthcoming 2022). 10.3389/pore.2022.1610373 PMC9284216 35845921

[93] Rubovszky G Kocsis J Boér K Chilingirova N Dank M Kahán Z Systemic Treatment of Breast Cancer. 1st Central-Eastern European Professional Consensus Statement on Breast Cancer. Pathol Oncol Res (Forthcoming 2022). 10.3389/pore.2022.1610383 PMC9311257 35898593

[94] Polgár C Kahán Z Ivanov O Chorváth M Ligačová A Csejtei A Radiotherapy of Breast Cancer – Professional Guideline. 1st Central-Eastern European Professional Consensus Statement on Breast Cancer. Pathol Oncol Res (Forthcoming 2022). 10.3389/pore.2022.1610378 PMC9272418 35832115

[95] Kahán Z Szántó I Dudás R Kapitány Z Molnár M Koncz Z Breast Cancer: Follow-Up, Rehabilitation, Psycho-Oncology. 1st Central-Eastern European Professional Consensus Statement on Breast Cancer. Pathol Oncol Res (Forthcoming 2022). 10.3389/pore.2022.1610391 PMC9200958 35721327

